# Supplementary material for: Fusion transcripts FYN-TRAF3IP2 and KHDRBS1-LCK hijack T cell receptor signaling in peripheral T-cell lymphoma, not otherwise specified
Source: Nat Commun. 2021 Jun 17;12:3705. doi: 10.1038/s41467-021-24037-4 (PMC8211700; doi:10.1038/s41467-021-24037-4)
Supplement: Supplementary file 1 — Supplementary Information [file 41467_2021_24037_MOESM1_ESM.pdf]

Supplementary information

Fusion transcripts FYN-TRAF3IP2 and KHDRBS1-LCK hijack T cell receptor signaling in peripheral T-cell lymphoma, not otherwise specified

Koen Debackere, Lukas Marcelis, Sofie Demeyer, Marlies Vanden Bempt, Nicole Mentens, Olga Gielen, Kris Jacobs, Michael Broux, Gregor Verhoef, Lucienne Michaux, Carlos Graux[7], Iwona Wlodarska, Philippe Gaulard, Laurence de Leval, Thomas Tousseyn, Jan Cools, Daan Dierickx

SUPPLEMENTARY FIGURES

Supplementary Figure 1

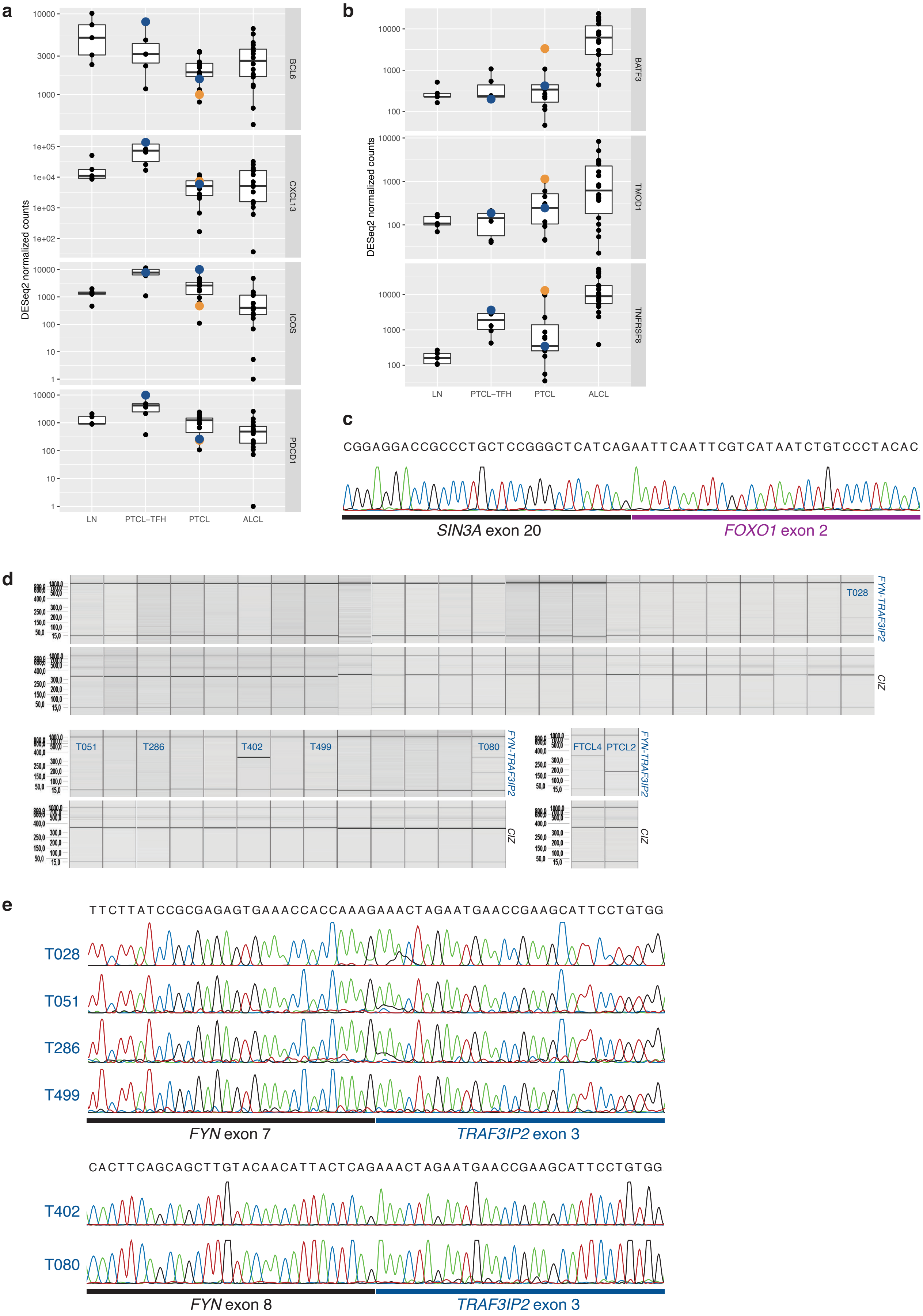

Supplementary Figure 1. Characterization of PTCL-NOS and PTCL-TFH cases. a Expression of T_{fh} markers in PTCL-NOS (PTCL) (*n* = 15), PTCL-TFH (*n* = 6) and ALCL cases (*n* = 18) and normal lymph nodes (LN) (*n* = 5). Blue dots represent the cases with a *FYN-TRAF3IP2* gene fusion. The orange dot represents the case with a *KHDRBS1-LCK* gene fusion. **b** Expression of *TNFRSF8*, *BATF3* and *TMOD1* in PTCL-NOS (PTCL) (*n* = 15), PTCL-TFH (*n* = 6) and ALCL cases (*n* = 18) and normal lymph nodes (LN) (*n* = 5). Blue dots represent the cases with a *FYN-TRAF3IP2* gene fusion. The orange dot represents the case with a *KHDRBS1-LCK* gene fusion. **c** Sanger sequencing of the RT-PCR amplicon for the *SIN3A-FOXO1* fusion in case PTCL14. **d** Gel separation pictures of the RT-PCR amplicons for the *FYN-TRAF3IP2* fusion transcript and *CIZ* control reaction for the validation cohort. **e** Sanger sequencing of the RT-PCR amplicons from the positive RT-PCR reactions for the *FYN-TRAF3IP2* fusion transcript in the validation cohort. For boxplots (**a-b**): the lower and upper hinges correspond to the first and third quartiles, respectively. The upper whisker extends from the hinge to the largest value no further than 1.5 times the interquartile range (IQR) from the hinge. The lower whisker extends from the hinge to the smallest value no further than 1.5 times the IQR from the hinge. Data beyond the end of the whiskers are outlying.

Supplementary Figure 2

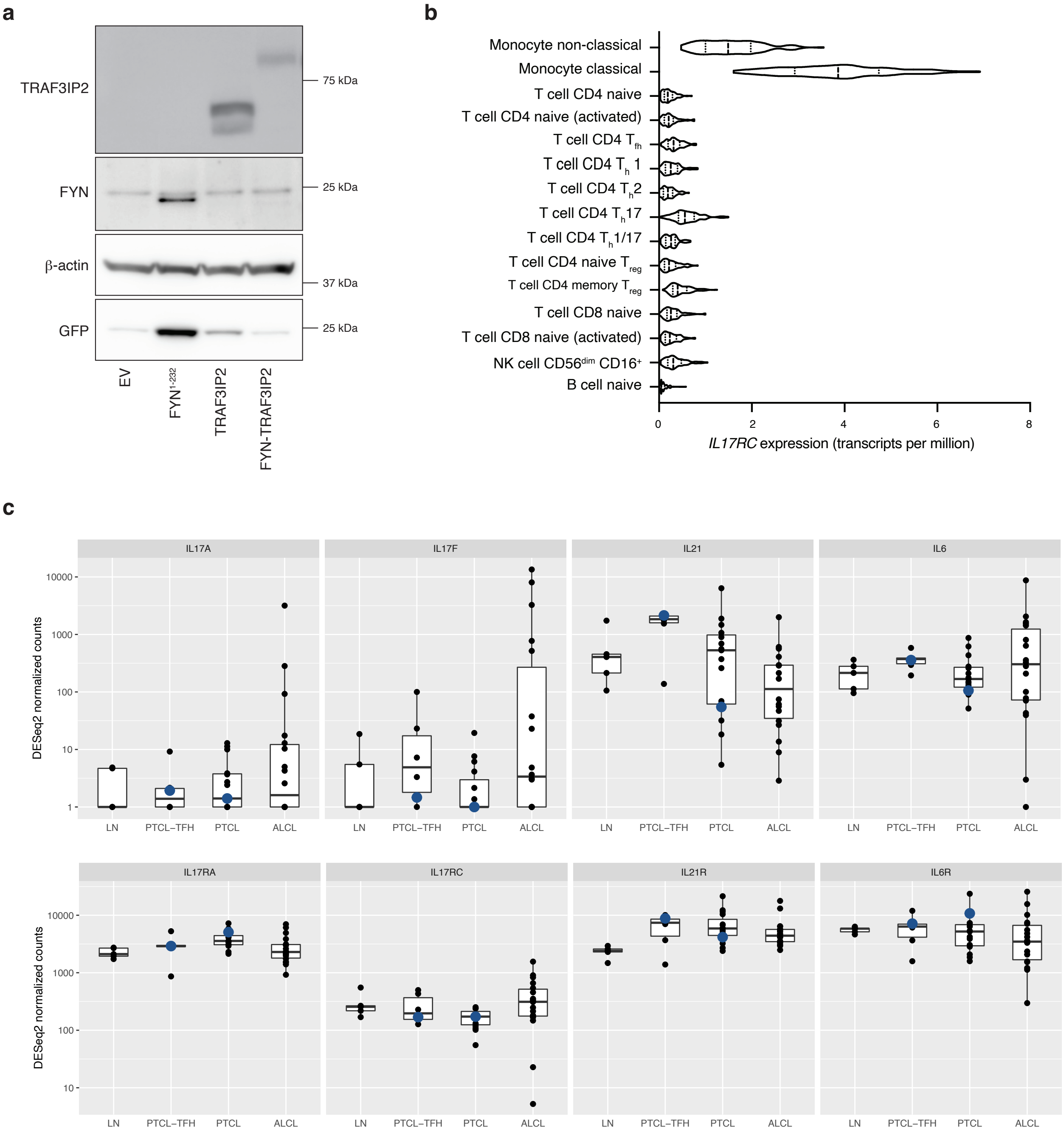

Supplementary Figure 2. Expression of pMIG constructs in Ba/F3 cells and expression of IL-17 signaling components in clinical samples. a Western blot for TRAF3IP2 and FYN in transduced Ba/F3 cells. **b** Expression of *IL17RC* in various cells from lymphoid and myeloid lineages from healthy volunteers. Data from DICE database. Dashed lines represent the median and dotted lines represent the lower and upper quartiles in the violin plots. **c**

Expression levels of *IL17A* and *IL17F* compared to expression levels of *IL21* and *IL6* in normal lymph nodes (LN) (*n* = 5), PTCL-TFH (*n* = 6), PTCL-NOS (PTCL) (*n* = 15) and ALCL (*n* = 18) (top). Expression levels of *IL17RC* compared to expression levels of *IL17RA*, *IL21R* and *IL6R* in normal lymph nodes (LN) (*n* = 5), PTCL-TFH (*n* = 6), PTCL-NOS (PTCL) (*n* = 15) and ALCL (*n* = 18) (bottom). The lower and upper hinges of the boxplots correspond to the first and third quartiles, respectively. The upper whisker extends from the hinge to the largest value no further than 1.5 times the IQR from the hinge. The lower whisker extends from the hinge to the smallest value no further than 1.5 times the IQR from the hinge. Data beyond the end of the whiskers are outlying.

Supplementary Figure 3

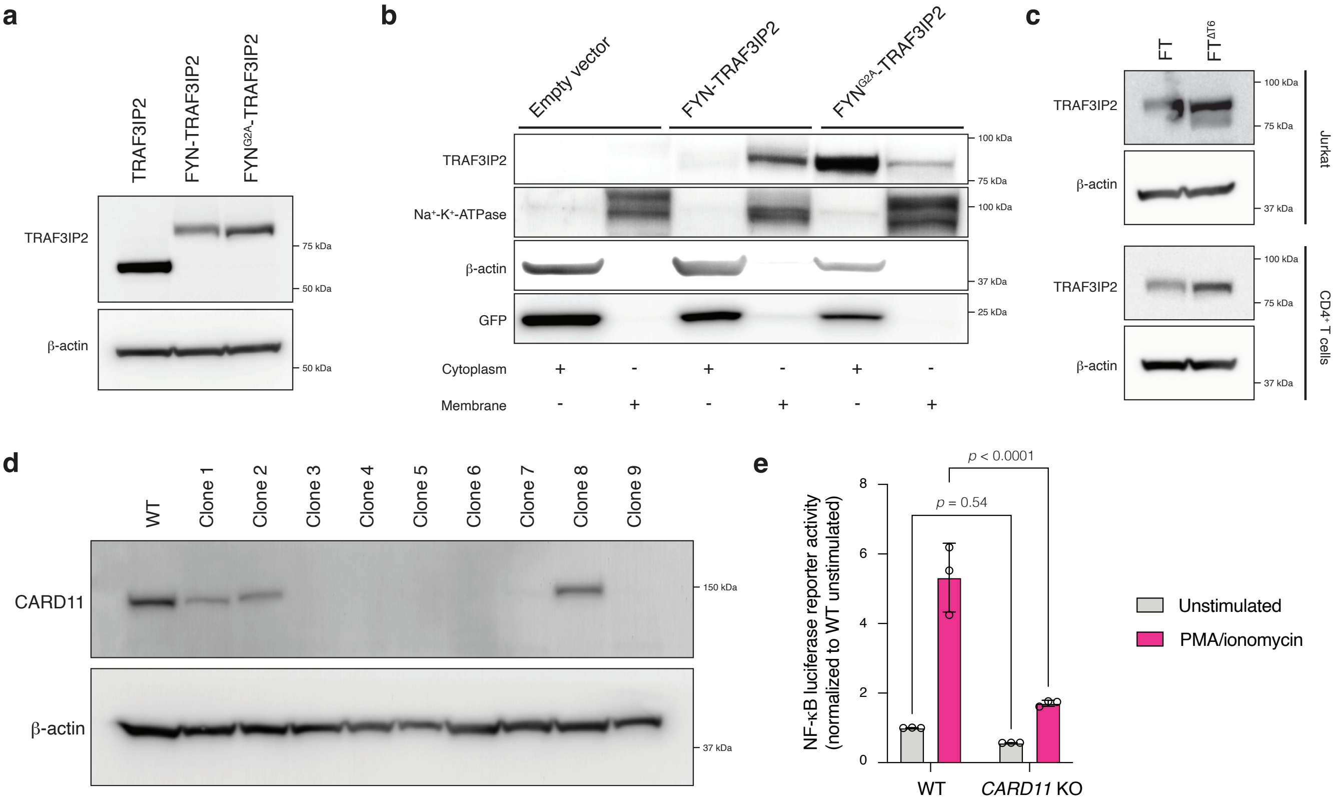

Supplementary Figure 3. Expression of pMIG constructs in Ba/F3 cells and CD4⁺ T cells and generation of *CARD11* knock-out Jurkat cells. a Western blot for TRAF3IP2 on lysates from CD4⁺ T cells transduced with pMIG-*TRAF3IP2*, pMIG-*FYN-TRAF3IP2* or pMIG-*FYN^{G2A}-TRAF3IP2*. **b** Western blot for TRAF3IP2 in the cytosolic and membrane fractions of Ba/F3 cells transduced with empty pMIG vector, pMIG-*FYN-TRAF3IP2* or pMIG-*FYN^{G2A}-TRAF3IP2*. **c** Western blot for TRAF3IP2 on lysates from Jurkat cells (top) and CD4⁺ T cells (bottom) transduced with pMIG-*FYN-TRAF3IP2* (FT) or pMIG-*FYN-TRAF3IP2^{ΔT6}* (FT^{ΔT6}). **d** Western blot for CARD11 on single-cell-derived Jurkat clones after electroporation with pX330 vector containing a sgRNA directed against *CARD11*. **e** NF-kB luciferase reporter activity in wild-type and *CARD11* knock-out Jurkat cells in basal conditions or after stimulation with

PMA/ionomycin. *n* = 3 replicates per condition. Data are represented as mean ± SD. *p*-values were calculated with Šidák's multiple comparisons test (two-sided).

Supplementary Figure 4

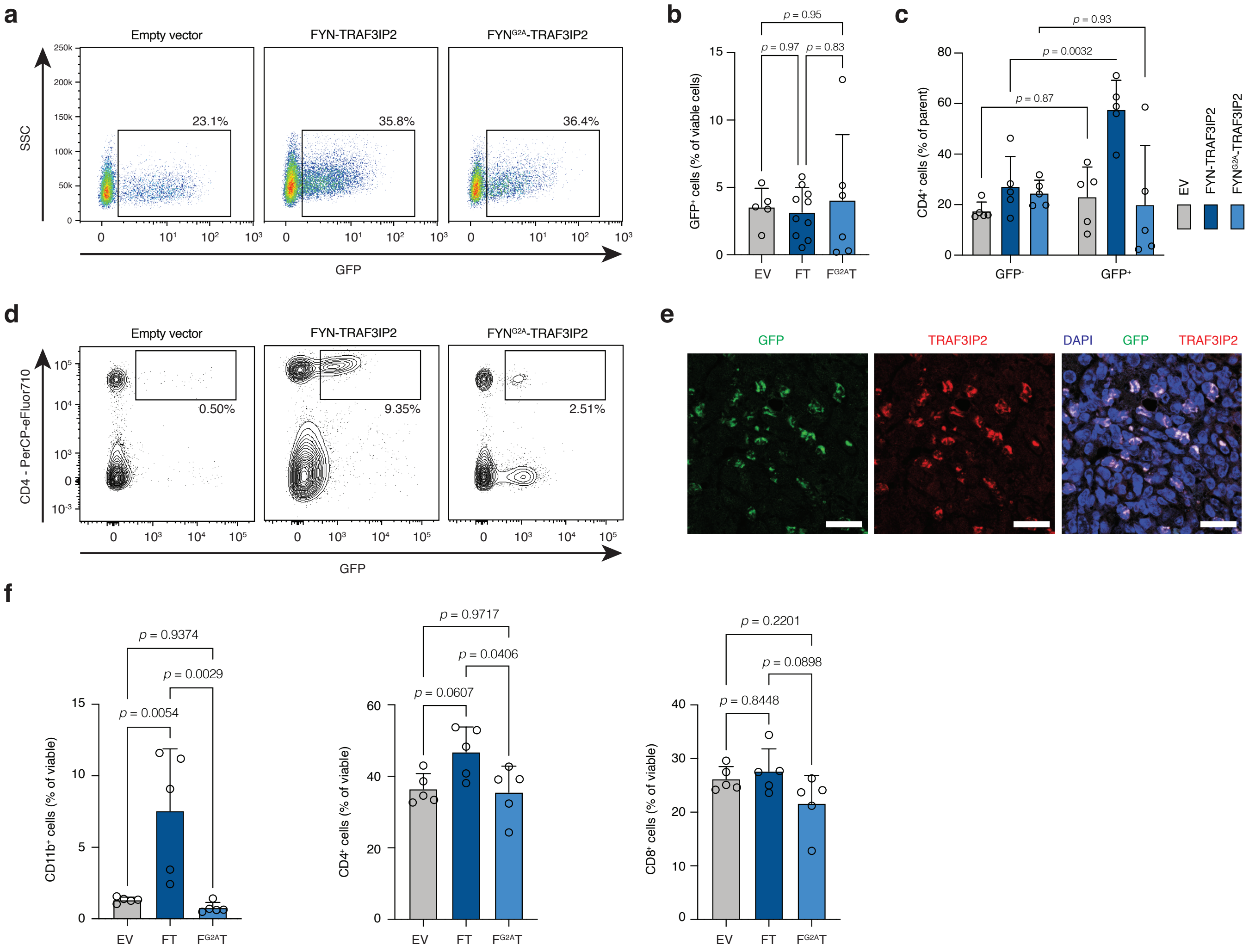

Supplementary Figure 4. Generation and phenotyping of *FYN-TRAF3IP2*-induced mouse lymphomas. a Transduction efficiency of HSPC 24 hours after retroviral transduction. **b** Quantification of GFP$^+$ cells in the peripheral blood 7 weeks after the bone marrow transplant procedure with HPSC transduced with empty vector (EV), *FYN-TRAF3IP2* (FT) *or FYN^{G2A}-TRAF3IP2* (F^{G2A}T). *n* = 5 mice for EV, *n* = 10 mice for FT and *n* = 6 mice for F^{G2A}T. *p*-values were calculated with Tukey's multiple comparisons test. **c** Quantification of CD4$^+$ T cells as a fraction of GFP$^-$ and GFP$^+$ cells in the peripheral blood 7 weeks after the bone marrow transplant procedure. *n* = 5 mice per group. *p*-values were calculated with Šidák's multiple comparisons

test (two-sided). **d** Representative flow cytometry plots for cell suspensions from the spleens of mice transplanted with HSPC transduced with empty vector, *FYN-TRAF3IP2* or *FYN*^{G2A}-*TRAF3IP2*. *n* = 5 mice per group. **e** Representative images of immunofluorescent staining for GFP and TRAF3IP2 on lymph node sections from *FYN-TRAF3IP2*-induced lymphomas (*n* = 5 mice). Scalebars represent 20 µm. **f** Quantification of CD11b$^+$ cells (left), CD4$^+$ T cells (middle) and CD8$^+$ T cells (right) in lymph node suspensions from mice transplanted with empty-vector-transduced (EV) cells, *FYN-TRAF3IP2*-transduced (FT) cells or *FYN*^{G2A}-*TRAF3IP2*-transduced cells (FG2AT). *n* = 5 mice per group. *p*-values were calculated with Tukey's post-hoc multiple comparisons test.

All data are represented as mean ± SD

Supplementary Figure 5

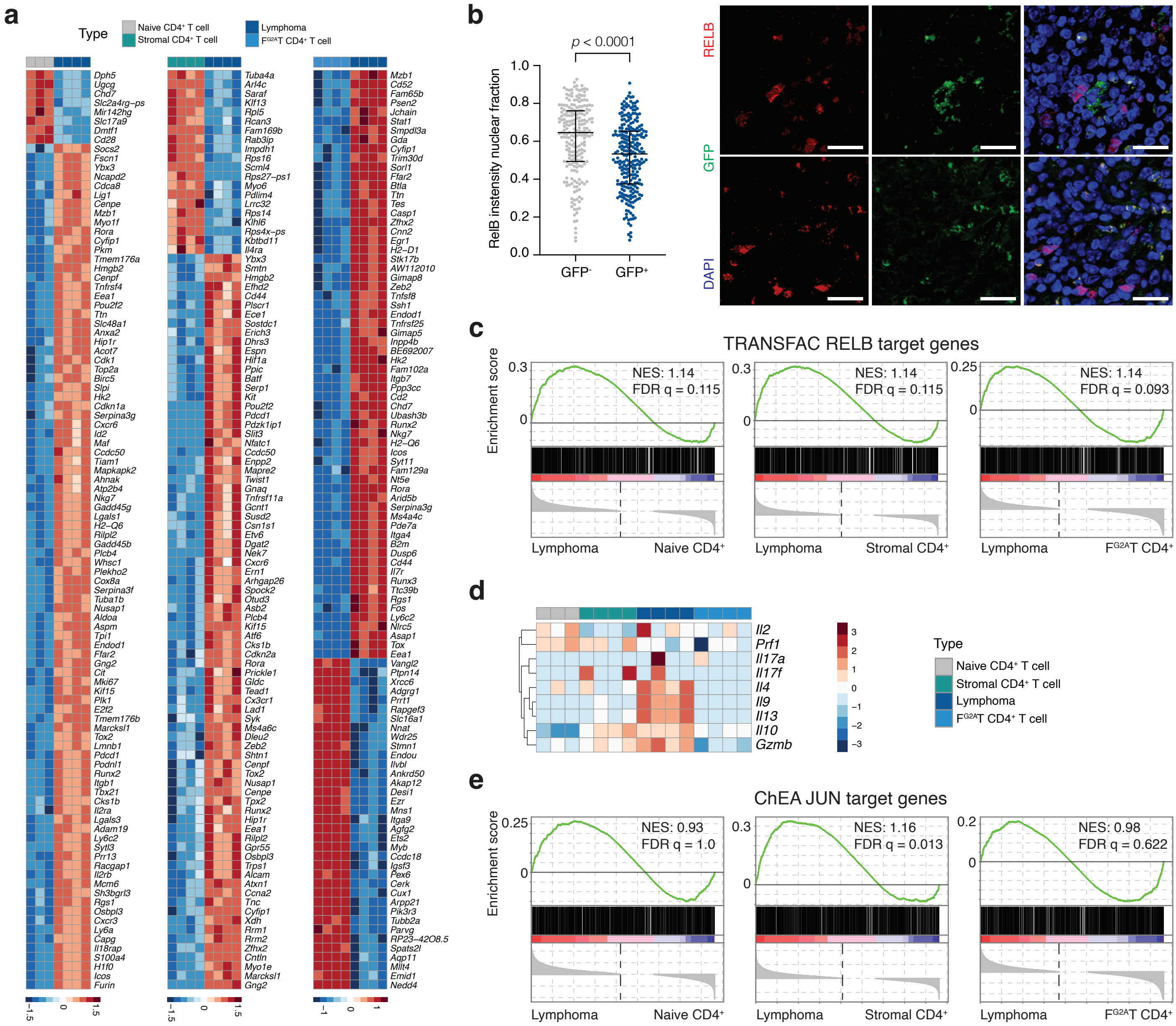

Supplementary Figure 5. Transcriptional profiling of *FYN-TRAF3IP2*-driven lymphomas. a Heatmaps for the most significant differentially expressed genes in CD4$^+$GFP$^+$ lymphoma cells versus naive CD4$^+$ T cells (left), CD4$^+$GFP$^-$ stromal T cells (middle) and CD4$^+$GFP$^+$ *FYN^{G2A}-TRAF3IP2 (F^{G2A}T)*-expressing T cells (right). Color scales represent the distribution for each row as mean ± SD. **b** Quantification (left) and representative immunofluorescent images (right) of the nuclear accumulation of RelB in GFP$^-$ stromal cells (n = 217 cells) and GFP$^+$

lymphoma cells (n = 257 cells) in the lymph nodes from mice (n = 5) with *FYN-TRAF3IP2*-induced lymphomas. Scalebars represent 20 µm. Horizontal line and whiskers represent median and interquartile range respectively. p-values were calculated with a two-sided Mann-Whitney test. **c** Enrichment plots for a list of computationally predicted RelB target genes in lymphoma cells compared with naive $CD4^+$ T cells (left), $CD4^+GFP^-$ stromal T cells (middle) and $CD4^+GFP^+$ *FYN*^{G2A}-*TRAF3IP2*-expressing T cells (right). **d** Heatmap representation of relative transcript abundance of T_h2-, T_h9- and T_h17-related cytokines and cytotoxic markers in naive $CD4^+$ T cells, $CD4^+GFP^-$ stromal T cells, *FYN-TRAF3IP2*-expressing lymphoma cells and $CD4^+GFP^+$ *FYN*^{G2A}-*TRAF3IP2*-expressing T cells. The color scale represents the distribution for each row as mean ± SD. **e** Enrichment plots for a list of c-Jun target genes identified by ChIP-seq in lymphoma cells compared with naive $CD4^+$ T cells (left), $CD4^+GFP^-$ stromal T cells (middle) and $CD4^+GFP^+$ *FYN*^{G2A}-*TRAF3IP2*-expressing T cells (right).

Supplementary Figure 6

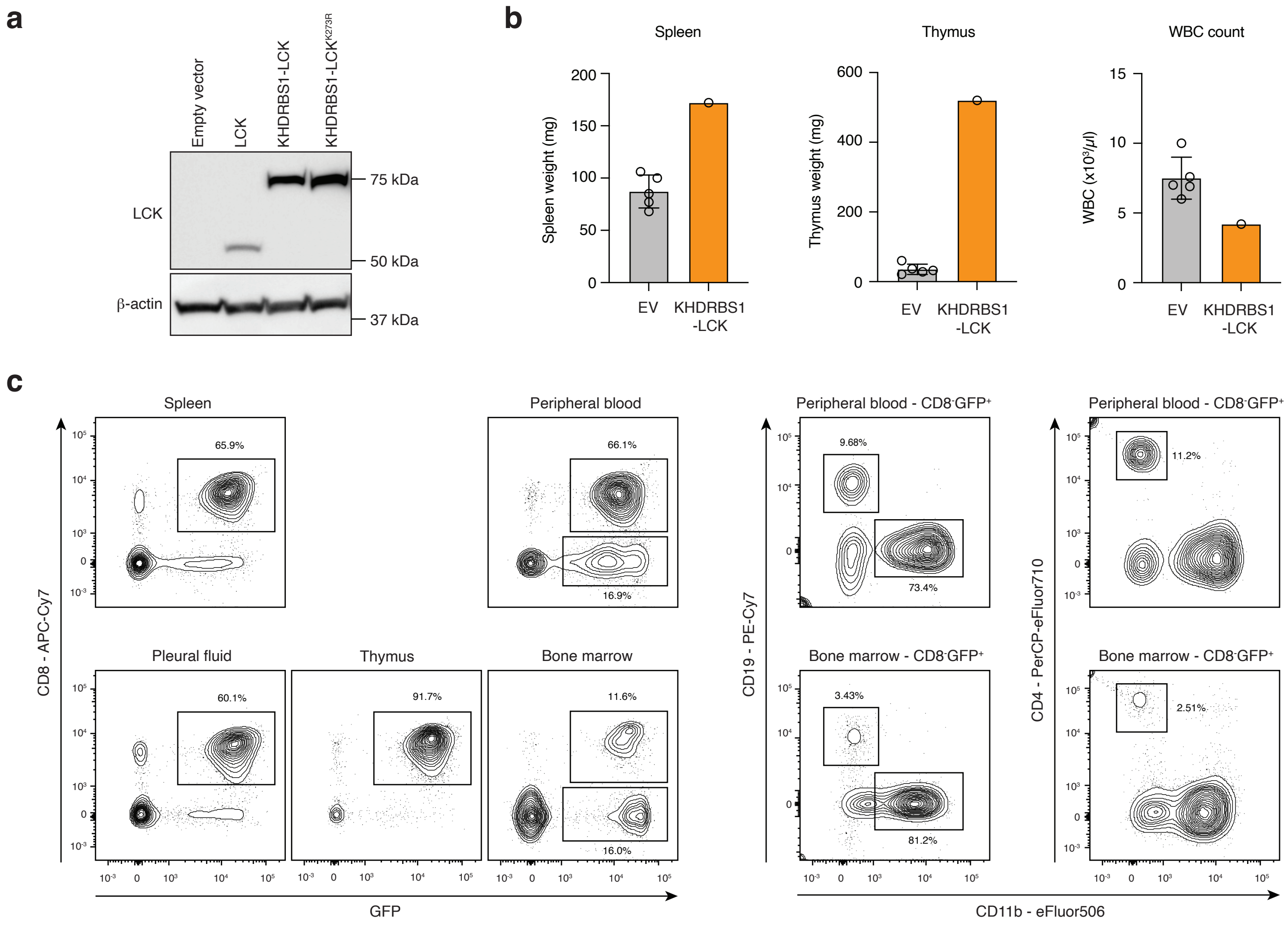

Supplementary Figure 6. Characterization of *KHDRBS1-LCK*-induced PTCL. a Western blot for LCK in Ba/F3 cells transduced with the indicated constructs. **b** Quantification of spleen weight, thymus weight and white blood cell (WBC) count in mice transplanted with HSPC transduced with empty pMIG vector (EV) or pMIG-*KHDRBS1-LCK*. n = 5 mice for EV, n = 1 mouse for KL. Data for EV are represented as mean ± SD. **c** Immunophenotype of GFP^{+} cells in the spleen, pleural fluid, thymus, peripheral blood and bone marrow of *KHDRBS1-LCK*-induced PTCL.

Supplementary Figure 7

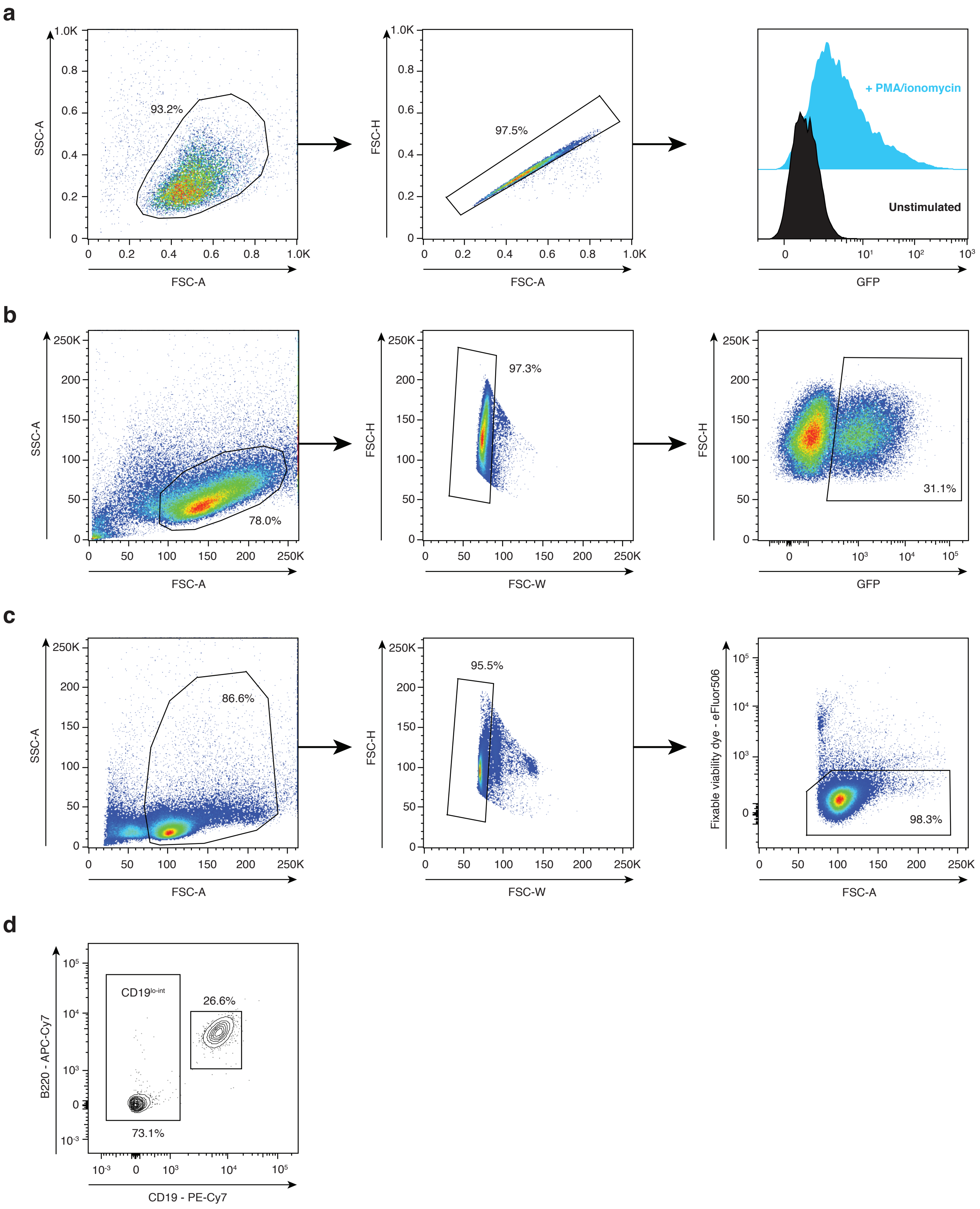

Supplementary Figure 7. Overview of gating strategies for flow cytometry. a Gating strategy for Jurkat NF-kB GFP reporter cells related to figures 2d, 3d. **b** Gating strategy for intracellular flow cytometry experiments related to figures 2f-g, 3f, 4e, 9e-f. **c** Gating strategy for cell suspensions from mouse tissues related to figures 5d, 6a, 6c, 10c-d and supplementary figures 3b-d, 3f. **d** Gating strategy for $CD19^{lo\text{-}int}$ cells related to figure 6e.

SUPPLEMENTARY TABLES

Supplementary Table 1

Case	Stage	IPI	Prior therapy	CD3	CD4	CD8	GZMB	PFN	TIA1	CD30	ICOS	PD1	EBV	*TET2*	*IDH2*	*DNMT3A*	*RHOA*	*FYN-TRAF3IP2*	*KHDRBS1-LCK*	*VAV1-MYO1F*	*TBL1XR1-TP63*	*SIN3A-FOXO1*
PTCL1	4	5	0	1	1	0	na	na	1	1	0	0	0	0	0	0	0	0	0	0	0	0
PTCL2	4	2	0	1	1	0	na	na	na	0	1	0	na	0	0	0	0	1	0	0	0	0
PTCL3	3	3	0	1	1	0	na	na	na	0	0	0	0	0	0	0	0	0	0	1	0	0
PTCL5	3	3	0	0	1	0	1	1	0	1	na	na	0	p.S1290L	0	0	0	0	0	0	0	0
PTCL9	2	1	0	1	1	0	0	0	0	1	na	0	na	0	0	0	0	0	0	0	0	0
PTCL11	1E	1	1	1	1	0	0	0	0	1	0	1	0	0	0	0	0	0	0	0	0	0
PTCL13	3	2	0	1	0	0	0	0	1	0	na	0	0	p.C1289R	p.R172S	0	0	0	0	0	0	0
PTCL14	3	2	0	1	0	1	1	1	1	0	na	na	0	p.Q196X, p.Q1034X	0	0	0	0	0	0	0	1
PTCL16	4	5	0	1	1	0	na	na	na	0	0	0	0	p.E1483X	0	p.D712fs	p.F30S	0	0	0	0	0
PTCL17	3	2	0	1	1	0	0	0	0	1	na	0	0	0	0	0	0	0	1	0	0	0
PTCL18	4	3	1	1	1	0	na	na	na	0	0	0	0	p.R1216X	0	0	0	0	0	0	0	0
PTCL19	4	4	0	1	0	0	na	1	na	1	na	na	0	0	0	p.G413fs	0	0	0	0	0	0
PTCL20	2	0	0	1	1	0	na	na	na	0	0	0	0	0	0	p.S337P	0	0	0	0	0	0
PTCL21	4	2	0	1	1	0	na	na	1	0	na	na	0	0	0	0	0	0	0	0	0	0
PTCL22	4	2	0	1	1	1	na	na	0	1	na	na	na	0	0	0	0	0	0	0	1	0
FTCL4	3	2	0	1	1	0	na	na	na	na	1	1	1	p.Q769X, p.W1219fs	p.R172K	p.R771P	p.G17V	1	0	0	0	0
FTCL5	3S	1	0	1	1	0	na	na	0	na	1	1	1	p.N1714fs, p.K1752fs	p.R172S	0	p.G17V	0	0	0	0	0
FTCL6	3S	4	0	1	1	0	na	na	na	na	1	1	1	p.Y1294C	p.R172M	0	p.G17V	0	0	0	0	0

Supplementary Table 1. Clinical, histopathological and genetic characteristics of the discovery cohort. M: male, F: female, IPI: international prognostic index, 0: absent, 1: present, na: not available.

Supplementary Table 2

FYN-TRAF3IP2	Sample ID	DIAGNOSIS		CD3	CD4	CD8	CD30	ICOS	PD1	CXCL13	BCL6	CD10	PFN	TIA1	GZMB
	T087	PTCL-NOS	non-TFH, non-cytotoxic	0	3	0	3	0	0	0	0	0	0	0	0
	T442	PTCL-NOS	non-TFH	3	0	0	2	3	0	0	0	0	n.a.	n.a.	n.a.
	T868	PTCL-NOS	cytotoxic	3	3	0	1	n.a.	n.a.	n.a.	n.a.	n.a.	1	3	1
	T1180	PTCL-NOS	unspecified	3	3	0	0	n.a.	3	n.a.	n.a.	n.a.	0	0	0
	T430	PTCL-NOS	cytotoxic	3	0	3	1	0	n.a.	0	0	0	n.a.	2	0
	T017	PTCL-NOS	unspecified	3	3	0	0	0	N.A.	N.A.	0	0	n.a.	0	n.a.
	T1091	PTCL-NOS	unspecified	3	3	0	1	0	N.A.	0	3	0	n.a.	n.a.	0
	T465	PTCL-NOS	unspecified	3	3	0	0	3	N.A.	0	0	0	1	n.a.	0
	T025	PTCL-NOS	unspecified	3	3	0	2	n.a.	0	0	3	0	0	0	n.a.
	T026	PTCL-NOS	unspecified	3	n.a.	0	1	n.a.	n.a.	n.a.	n.a.	n.a.	n.a.	n.a.	n.a.
	T425	PTCL-NOS	TFH	3	n.a.	n.a.	N.A.	3	n.a.	0	1	0	n.a.	n.a.	n.a.
	T514	PTCL-NOS	TFH	3	3	0	2	3	n.a.	0	2	0	n.a.	n.a.	n.a.
	T376	PTCL-NOS	unspecified	3	3	0	n.a.	3	n.a.	n.a.	n.a.	0	0	0	n.a.
	T493	PTCL-NOS	unspecified	3	3	0	n.a.	n.a.	2	n.a.	n.a.	n.a.	n.a.	n.a.	n.a.
	T513	PTCL-NOS	TFH	3	n.a.	n.a.	n.a.	1	0	n.a.	0	1	n.a.	n.a.	n.a.
yes	T028	PTCL-NOS	cytotoxic	3	3	0	0	n.a.	n.a.	n.a.	n.a.	n.a.	0	2	0
yes	T051	PTCL-NOS	TFH	3	n.a.	0	n.a.	n.a.	3	1	n.a.	1	n.a.	0	0
	T117	PTCL-NOS	unspecified	1	n.a.	0	0	0	0	n.a.	n.a.	n.a.	0	0	0
yes	T286	PTCL-NOS	TFH	3	3	0	n.a.	3	0	1	0	0	n.a.	n.a.	n.a.
	T309	PTCL-NOS	unspecified	3	2	0	0	0	n.a.	n.a.	n.a.	0	n.a.	0	0
	T326	PTCL-NOS	cytotoxic	3	n.a.	0	1	3	0	0	0	0	0	3	0
yes	T402	PTCL-NOS	cytotoxic	2	0	0	0	n.a.	0	0	0	n.a.	2	0	3
	T423	PTCL-NOS	cytotoxic	2	0	2	0	n.a.	n.a.	n.a.	n.a.	n.a.	2	2	2
yes	T499	PTCL-NOS	unspecified (but RHOA p.G17V)	3	3	0	1	n.a.	n.a.	n.a.	n.a.	n.a.	0	n.a.	0
	T543	ATLL		3	3	0	0	0	n.a.	1	n.a.	n.a.	n.a.	0	n.a.
	T594	PTCL-NOS	cytotoxic	3	0	3	2	n.a.	0	0	1	0	3	3	n.a.
	T019	PTCL-NOS	unspecified	2	0	0	3	0	0	0	0	0	0	0	0
	T079	PTCL-NOS	cytotoxic	0	3	0	1	0	0	0	0	0	3	3	n.a.
yes	T080	PTCL-NOS	cytotoxic	3	0	3	3	0	n.a.	n.a.	n.a.	0	3	3	3
	T083	PTCL-NOS	cytotoxic	0	3	0	3	0	n.a.	n.a.	n.a.	n.a.	3	3	n.a.
	T103	PTCL-NOS	unspecified	2	n.a.	0	3	3	n.a.	0	0	0	0	0	0
	T149	PTCL-NOS	cytotoxic	3	n.a.	3	3	n.a.	1	n.a.	0	0	n.a.	1	n.a.
	T150	PTCL-NOS	cytotoxic	3	0	3	0	n.a.	n.a.	0	n.a.	0	n.a.	3	3
	T153	PTCL-NOS	unspecified	3	n.a.	n.a.	0	3	0	0	n.a.	0	0	0	n.a.
	T173	PTCL-NOS	unspecified	0	n.a.	3	3	n.a.	0	n.a.	n.a.	1	n.a.	n.a.	n.a.
	T177	PTCL-NOS	unspecified	3	3	0	3	0	0	0	0	0	n.a.	0	0
	T306	PTCL-NOS	unspecified	0	2	0	2	n.a.	n.a.	n.a.	n.a.	0	0	0	1

Supplementary Table 2. Histopathological characteristics of the validation cohort. Samples with a FYN-TRAF3IP2 gene fusion are marked indicated in the first column. The second column contains the sample name, the third column contains the histopathological diagnosis. The fourth to final column contain the results of immunohistochemical for the markers indicated in the column headers. ATLL: adult T-cell Leukemia/Lymphoma, green box: absent, red box: present, 0: negative, 1: weak, 2: moderate, 3: strong, n.a.: not available.

Supplementary Table 3

Name	Sequence
**FYN-TRAF3IP2 breakpoint Fwd**	CGAAAAGATGCTGAGCGACA
**FYN-TRAF3IP2 breakpoint exon 8 Fwd**	TGAAAGGAGACCATGTCAAACA
**FYN-TRAF3IP2 breakpoint Rev**	TTCTGATTCCTCTTCCGGGG
**TRAF3IP2 EcoRI-Kozak-start**	CCGGAATTCGCCACCATGAACCGAAGCATTCCTGTG
**FYN-TRAF3IP2 EcoRI-Kozak-start**	CCGGAATTCGCCACCATGGGCTGTGTGCAATGTAA
**FYN(G2A)-TRAF3IP2 EcoRI-Kozak-start**	CCGGAATTCGCCACCATGGCCTGTGTGCAATGTAAG
**FYN-TRAF3IP2 stop-BamHI**	CGCGGATCCTCACAAGGGAACCACCTGAA
**FYN$^{1\text{-}232}$ stop-BamHI**	CGCGGATCCTCATGAGTAATGTTGTACAAGCTGCTG
**FYN-TRAF3IP2 Q5SDM ΔT6 Fwd**	gttgctgcaTCAGAACCATACCCAAGTC
**FYN-TRAF3IP2 Q5SDM ΔT6 Rev**	cgccacaggAATGCTTCGGTTCATTCTAG
**gDNA FYN nested outer**	GCAAGCTCAGCCATTCATCG
**gDNA FYN nested inner**	AGCTTTCAGTCTCAAGGGCA
**gDNA TRAF3IP2 nested outer**	GGGAAAGGCCTGAAGATGCT
**gDNA TRAF3IP2 nested inner**	CACGGGAGAGCAACTCTTCA
**LCK EcoRI-Kozak-start**	CCGGAATTCGCCACCATGGGCTGTGGCTGCAG
**KHDRBS1-LCK breakpoint Fwd**	GCTGACGGCAGAAATTGAGA
**KHDRBS1-LCK breakpoint Rev**	TGTTCCCCCTTCTCAAAGCC
**KHDRBS1-LCK EcoRI-Kozak-start**	CCGGAATTCGCCACCATGCAGCGCCGGGACGAC
**KHDRBS1-LCK stop-XhoI**	CCGCTCGAGTCAAGGCTGAGGCTGGTACTG
**KHDRBS1-LCK Q5SDM K273R Fwd**	GTGGCGGTGAgGAGCCTGAAG
**KHDRBS1-LCK Q5SDM K273R Rev**	CTTCGTGTGCCCGTTGTAG
**SIN3A-FOXO1 breakpoint Fwd**	GCCTGAGCTTCGTGAACAT
**SIN3A-FOXO1 breakpoint Rev**	ACTGTGATCCAGGGCTGTC
**Trbv5 Fwd**	GGAAACAGCACTCATGAACAC
**Trbv1 Fwd**	CTACAGACCCCACAGTGAC
**Trbv26 Fwd**	CAAGATATCTGGTGAAAGGGC
**Trbv2 Fwd**	AGTATCTAGGCCACAATGC
**Trbv12-1/12-2/12-3 Fwd**	CTCYTGGGAACAAGTTCAGC
**Trbv19 Fwd**	CACATGGTGATGGTGGCATC
**Trbv29 Fwd**	GAACAGGCCTTGTGGACATG
**Trbv13-1/13-2/13-3 Fwd**	TGKGWRCAAAACACATGGAGGC
**Trbv17 Fwd**	TGCAGCCACTTTTGTGGATAC
**Trbv4 Fwd**	AATTGCTGAAGATTATGTTTAGC
**Trbv16 Fwd**	GAGAGCAGAACCAACAAATGC
**Trbv15 Fwd**	GCAAGTCTCTTATGGAAGATGG
**Trbv14 Fwd**	GTTCCTTGACACAGTACTGTC
**Trbv31 Fwd**	GCTCAGACTATCCATCAATGG
**Trbv20 Fwd**	TTCTGGGGCCTGGCTGTG
**Trbv3 Fwd**	GAAGCAGGACACACAGGAC

**Trbv18 Fwd**	GCAGCTCTTTATGTTGCTGG
**Trbv30 Fwd**	TGTCCTCCTCTACCAAAAGC
**Trbv21 Fwd**	CTCTGGGGTTGTCCAGAATC
**Trbc1/Trbc2 Rev**	CTATAATTGCTCTCCTTGTAGG
**Ciz Fwd**	CCCATTTCGGCTCCCATGATT
**Ciz Rev**	GGTCTCGGTGTGTGACTTGGA
**Arid5a Fwd**	TCATGAAGGAGCGACACACG
**Arid5a Rev**	CCTGTCACCAGCTCATAGGC
**Zc3h12a Fwd**	AACTGGTTTCTGGAGCGAGG
**Zc3h12a rev**	CGAAGGATGTGCTGGTCTGT
**Bcl2l1 Fwd**	GCCTTTTTCTCCTTTGGCGG
**Bcl2l1 Rev**	TCCACAAAAGTGTCCCAGCC
**CARD11 TS1 Fwd**	CACCGGACGGCCTGATCACATCGG
**CARD11 TS1 Rev**	AAACCCGATGTGATCAGGCCGTCC

Supplementary Table 3. Primers used in the study.

Supplementary Table 4

Antigen	Clone	Manufacturer	Catalog	Dilution
TRAF3IP2 (ACT1)	9ACT12	Thermo Fisher Scientific	14-4040-82	1/1000
FYN	15	Santa Cruz Biotechnologies	sc-434	1/200
p65	D14E12	Cell Signaling	8242S	1/2000
Phospho-p65 (Ser536)	93H1	Cell Signaling	3033S	1/1000
p100/p52	polyclonal	Cell Signaling	4882S	1/1000
Phospho-p100 (Ser866/870)	polyclonal	Cell Signaling	4810S	1/1000
ERK1/2	C-16	Santa Cruz Biotechnologies	sc-93	1/1000
Phospho-pERK1/2 (Thr202/Tyr204)	polyclonal	Cell Signaling	9101S	1/1000
JNK	polyclonal	Cell Signaling	9252S	1/1000
Phospho-JNK (Thr183/Tyr185)	G9	Cell Signaling	9255S	1/1000
p38	A-12	Santa Cruz Biotechnologies	sc-7972	1/1000
Phospho-p38 (Thr180/Tyr182)	3D7	Cell Signaling	9215S	1/1000
TRAF6	D21G3	Cell Signaling	8028S	1/1000
K63-linked polyubiquitin	D7A11	Cell Signaling	5621S	1/1000
CARD11	1D12	Cell Signaling	4435S	1/2000
LCK	73A5	Cell Signaling	2787S	1/1000
Phospho-LCK (Tyr394)	polyclonal	Sigma	SAB4300118	1/1000
Na,K-ATPASE	polyclonal	Cell Signaling	3010	1/1000
GFP	B-2	Santa Cruz Biotechnologies	sc-9996	1/1000
Beta-actin	AC-15	Sigma	A1978	1/5000
ECL Rat IgG, HRP-linked whole antibody	polyclonal	Cytiva	NA935	1/5000
ECL Rabbit IgG, HRP-linked whole antibody	polyclonal	Cytiva	NA934	1/5000
ECL Mouse IgG, HRP-linked whole antibody	polyclonal	Cytiva	NA931	1/5000

Supplementary Table 4. Antibodies used for Western blot.

Supplementary Table 5

Antigen	Clone	Manufacturer	Catalog number	Dilution
CD3E	17A2	Miltenyi Biotec	130-118-849	1/60
CD4	RM4-5	eBioscience	46-0042-82	1/400
CD4	RM4-5	eBioscience	45-0042-82	1/400
CD4	GK1.5	eBioscience	25-0041-81	1/400
CD4	GK1.5	eBioscience	17-0041-82	1/400
CD8	53-6.7	eBioscience	47-0081-82	1/200
CD11b	M1/70	Biolegend	101226	1/200
CD11b	M1/70	eBioscience	69-0112-82	1/200
CD11b	M1/70	eBioscience	12-0112-83	1/400
CD16/32	93	eBioscience	14-0161-86	18264
CD19	eBio1D3	eBioscience	25-0193-81	1/400
CD25	PC61.5	eBioscience	17-0251-82	1/400
CD44	IM7	BD Biosciences	560570	1/400
CD69	H1.2F3	eBioscience	25-0691-81	1/300
CD95	Jo2	BD Biosciences	562633	1/100
CD138	281-2	Biolegend	142503	1/200
CD185/CXCR5	L138D7	Biolegend	145506	1/100
CD278/ICOS	C398.4A	Biolegend	313508	1/100
CD278/ICOS	C398.4A	BD Biosciences	565885	1/100
CD279/PD-1	29F.1A12	Biolegend	135218	5 µl/test
CD279/PD-1	29F.1A12	Biolegend	135206	1/100
B220	RA3-6B2	Biolegend	103224	1/200
GL7	GL7	Biolegend	144617	1/200
Gr1	RB6-8C5	eBioscience	12-5931-82	1/500
Gr1	RB6-8C5	eBioscience	17-5931-82	1/400
TCRbeta	H57-597	eBioscience	12-5961-82	1/400
BCL6	K112-91	BD Biosciences	563363	5 µl/test
TdT	19-3	eBioscience	17-5846-82	1/200
Phospho-ERK1/2	MILAN8R	eBioscience	17-9109-42	5 µl/test
Phospho-JNK	N9-66	BD Biosciences	562480	5 µl/test
Phospho-p38	36/p38	BD Biosciences	612565	20 µl/test
Phospho-ZAP70	n3kobu5	eBioscience	25-9006-42	5 µl/test
Phospho-p65	93H1	Cell Signaling	3033S	1/1600
IkBa	E130	Abcam	ab32518	1/50
Donkey anti-rabbit IgG Alexa Fluor 647	n.a.	Invitrogen	A-31573	1/2500
Fixable viability dye eFluor 506	n.a.	eBioscience	65-0866-18	1/1000
Fixable viability dye eFluor 450	n.a.	eBioscience	65-0863-14	1/1000

Supplementary Table 5. Antibodies used for flow cytometry.
