# Supplementary material for: Fusion transcripts FYN-TRAF3IP2 and KHDRBS1-LCK hijack T cell receptor signaling in peripheral T-cell lymphoma, not otherwise specified
Source: Nat Commun. 2021 Jun 17;12:3705. doi: 10.1038/s41467-021-24037-4 (PMC8211700; doi:10.1038/s41467-021-24037-4)
Supplement: Supplementary file 3 — Reporting summary [file 41467_2021_24037_MOESM3_ESM.pdf]

nature research

Corresponding author(s): Jan Cools

Last updated by author(s): 2021/04/28

Reporting Summary

Nature Research wishes to improve the reproducibility of the work that we publish. This form provides structure for consistency and transparency in reporting. For further information on Nature Research policies, see our Editorial Policies and the Editorial Policy Checklist.

Statistics

For all statistical analyses, confirm that the following items are present in the figure legend, table legend, main text, or Methods section.

n/a	Confirmed	
☐	☒	The exact sample size (*n*) for each experimental group/condition, given as a discrete number and unit of measurement
☐	☒	A statement on whether measurements were taken from distinct samples or whether the same sample was measured repeatedly
☐	☒	The statistical test(s) used AND whether they are one- or two-sided<br>*Only common tests should be described solely by name; describe more complex techniques in the Methods section.*
☒	☐	A description of all covariates tested
☐	☒	A description of any assumptions or corrections, such as tests of normality and adjustment for multiple comparisons
☐	☒	A full description of the statistical parameters including central tendency (e.g. means) or other basic estimates (e.g. regression coefficient) AND variation (e.g. standard deviation) or associated estimates of uncertainty (e.g. confidence intervals)
☐	☒	For null hypothesis testing, the test statistic (e.g. *F*, *t*, *r*) with confidence intervals, effect sizes, degrees of freedom and *P* value noted<br>*Give P values as exact values whenever suitable.*
☒	☐	For Bayesian analysis, information on the choice of priors and Markov chain Monte Carlo settings
☒	☐	For hierarchical and complex designs, identification of the appropriate level for tests and full reporting of outcomes
☒	☐	Estimates of effect sizes (e.g. Cohen's *d*, Pearson's *r*), indicating how they were calculated

Our web collection on statistics for biologists contains articles on many of the points above.

Software and code

Policy information about availability of computer code

Data collection	Next generation sequencing was performed using NovaSeq Control Software v1.6 on the Illumina NovaSeq 6000 instrument and HiSeq Control Software v3.4.0 on the Illumina HiSeq 4000 instrument. We acquired flow cytometry data on a FACS Canto cytometer (BD Biosciences) using BD FACSDiva software v6.1.3, a FACSVerse cytometer (BD Biosciences) using BD FACSuite software v1.0.6, an LSRFortessa X-20 cytometer (BD Biosciences) using BD FACSDiva software v9.0, a MACSQuant Vyb cytometer (Miltenyi) using MACSQuantify software v2.11. Cells were sorted with an S3 cell sorter (Biorad) using ProSort v1.6 software or a Sony MA900 cell sorter using Sony Cell Sorter Software v3.0. PCR products were analyzed with a QIAxcel system (Qiagen) using BioCalculator software v3.2. qPCR was performed with a ViiiA7 instrument (Applied Biosystems) using ViiA7 software v1.2. Western blots were acquired on the ImageQuant LAS 4000 (GE Health Care) using ImageQuant LAS 4000 v1.3.0.134 software. Confocal images were acquired on the SP8 X confocal microscope (Leica) using Las X v2 software. H&E images and immunohistochemistry images were acquired with a Leica DMI6000B microscope (Leica) and a Vectra Polaris slide scanner (Akoya). We used a Victor X4 multilabel plate reader (Perkin Elmer) with WorkOut v2.5 software to acquire luminescence data for luciferase experiments and ATPlite (Perkin Elmer) cell viability assays.<br>The NF-kB target gene set was obtained from the Gilmore lab (https://www.bu.edu/nf-kb/gene-resources/target-genes/).<br>Other gene sets were obtained from Harmonizome (https://maayanlab.cloud/Harmonizome/)
Data analysis	The following software was used to analyze data:<br>- bcl2fastq v2.20<br>- FastQC v0.11.9<br>- fastq-MCF (ea-utils v1.1.2)<br>- HISAT2 v2.1.0<br>- SAMtools v1.10<br>- FusionCatcher v1.0<br>- HTSeq v0.9.1<br>- R v4.0

- DESeq2 v1.24.0
- GSEA v4.1.0
- i-cisTarget (https://gbiomed.kuleuven.be/apps/lcb/i-cisTarget/index.php)
- SLqPCR v1.54.0 (DOI 10.18129/B9.bioc.SLqPCR)
- FlowJo v10.6
- CellProfiler v4.0.6
- Graphpad Prism v9
- ggplot v3.3.2

No custom code was generated for this study.

For manuscripts utilizing custom algorithms or software that are central to the research but not yet described in published literature, software must be made available to editors and reviewers. We strongly encourage code deposition in a community repository (e.g. GitHub). See the Nature Research guidelines for submitting code & software for further information.

Data

Policy information about availability of data

All manuscripts must include a data availability statement. This statement should provide the following information, where applicable:

- Accession codes, unique identifiers, or web links for publicly available datasets
- A list of figures that have associated raw data
- A description of any restrictions on data availability

Raw sequence data from clinical specimens has been deposited in the European Genome-Phenome Archive (accession EGAS00001004646 for 3 PTCL-TFH cases, accession EGAS00001005015 for 15 PTCL-NOS cases). Raw sequence data from murine CD4+ T cells is available in the European Nucleotide Archive (accession PRJEB42764). Sequence data from anaplastic large cell lymphoma was obtained from the Sequence Read Archive (accession SRP044708) and sequence data from healthy lymph nodes was obtained from ArrayExpress (accession E-MTAB-2836). Expression data for immune cells in healthy individuals used in Supplementary figure 2b were obtained from DICE. The source data underlying Figures 2, 3, 4, 5, 6, 7, 8, 9, 10 and Supplementary Figures 1, 2, 3, 4, 5,6 are provided as a Source Data file. All the other data supporting the findings of this study are available within the article and its supplementary information files and from the corresponding author upon reasonable request.

Field-specific reporting

Please select the one below that is the best fit for your research. If you are not sure, read the appropriate sections before making your selection.

☒ Life sciences ☐ Behavioural & social sciences ☐ Ecological, evolutionary & environmental sciences

For a reference copy of the document with all sections, see nature.com/documents/nr-reporting-summary-flat.pdf

Life sciences study design

All studies must disclose on these points even when the disclosure is negative.

Sample size	- The number of patient samples was determined by the availability of samples.<br>- For studies with mice, we used at least 5 mice per group. This sample size is adequate disease alleles with high penetrance will cause disease in a majority of the animals. The sample size was chosen based on previous experience in our laboratory (e.g. PMID 29496663), sample size in literature in the field of mouse models of T cell lymphoma (e.g. PMID 21926465, PMID 29398449)
Data exclusions	In the KHDRBS1-LCK group, one mouse developed an ulcerated squamous cell carcinoma of the skin and was censored in the survival curve. No other data were excluded from the analyses.
Replication	All in vitro experiments used at least 3 biological independent replicates. In vitro experiments were reproduced at least twice with congruent results. We used different batches of virus for repetitions of experiments involving viral transduction. The number of biological replicates is included the figure legends. The number of repetitions for every experiment is included under the header Statistics and reproducibility in the Methods section of the main article.
Randomization	For drug treatments, drugs were dispensed randomly with a D300e digital dispenser (Tecan) in 96-well plates. For all other in vitro experiments, wells with identical number of cells were randomly allocated to each group.<br>For bone marrow transplant models, mice of the same age were randomly allocated to be injected with lineage negative cells transduced with different constructs. Mouse samples used for flow cytometry, immunohistochemistry and flow cytometry were chosen randomly.
Blinding	The thresholds for the analysis of nuclear NF-kB accumulation in mouse lymphomas, were set by a person unaware of the experimental hypothesis. For all other experiments, experimental setup and differences in treatment of samples precluded blinding. For the analysis of flow cytometry data (growth curves, tumor samples, phosphoflow), fixed gates were applied to analyze all samples within an experiment.

Reporting for specific materials, systems and methods

We require information from authors about some types of materials, experimental systems and methods used in many studies. Here, indicate whether each material, system or method listed is relevant to your study. If you are not sure if a list item applies to your research, read the appropriate section before selecting a response.

Materials & experimental systems

n/a	Involved in the study
	x Antibodies
	x Eukaryotic cell lines
x	Palaeontology and archaeology
	x Animals and other organisms
	x Human research participants
x	Clinical data
x	Dual use research of concern

Methods

n/a	Involved in the study
x	ChIP-seq
	x Flow cytometry
x	MRI-based neuroimaging

Antibodies

Antibodies used

ANTIBODIES FOR FLOW CYTOMETRY
Antigen, Clone , Manufacturer, Catalog number, Dilution
CD3E, 17A2, Miltenyi Biotec, 130-118-849, 1/60
CD4, RM4-5, eBioscience, 46-0042-82, 1/400
CD4, RM4-5, eBioscience, 45-0042-82, 1/400
CD4, GK1.5, eBioscience, 25-0041-81, 1/400
CD4, GK1.5, eBioscience, 17-0041-82, 1/400
CD8, 53-6.7, eBioscience, 47-0081-82, 1/200
CD11b, M1/70, Biolegend, 101226, 1/200
CD11b, M1/70, eBioscience, 69-0112-82, 1/200
CD11b, M1/70, eBioscience, 12-0112-83, 1/400
CD16/32, 93, eBioscience, 14-0161-86, 1/50
CD19, eBio1D3, eBioscience, 25-0193-81, 1/400
CD25, PC61.5, eBioscience, 17-0251-82, 1/400
CD44, IM7, BD Biosciences, 560570, 1/400
CD69, H1.2F3, eBioscience, 25-0691-81, 1/300
CD95, Jo2, BD Biosciences, 562633, 1/100
CD138, 281-2, Biolegend, 142503, 1/200
CD185/CXCR5, L138D7, Biolegend, 145506, 1/100
CD278/ICOS, C398.4A, Biolegend, 313508, 1/100
CD278/ICOS, C398.4A, BD Biosciences, 565885, 1/100
CD279/PD-1, 29F.1A12, Biolegend, 135218, 5 µl/test
CD279/PD-1, 29F.1A12, Biolegend, 135206, 1/100
B220, RA3-6B2, Biolegend, 103224, 1/200
GL7, GL7, Biolegend, 144617, 1/200
Gr1, RB6-8C5, eBioscience, 12-5931-82, 1/500
Gr1, RB6-8C5, eBioscience, 17-5931-82, 1/400
TCRbeta, H57-597, eBioscience, 12-5961-82, 1/400
BCL6, K112-91, BD Biosciences, 563363, 5 µl/test
TdT, 19-3, eBioscience, 17-5846-82, 1/200
Phospho-ERK1/2, MILAN8R, eBioscience, 17-9109-42, 5 µl/test
Phospho-JNK, N9-66, BD Biosciences, 562480, 5 µl/test
Phospho-p38, 36/p38, BD Biosciences, 612565, 20 µl/test
Phospho-ZAP70, n3kobu5, eBioscience, 25-9006-42, 5 µl/test
Phospho-p65, 93H1, Cell Signaling, 3033S, 1/1600
IkBa, E130, Abcam, ab32518, 1/50
Donkey anti-rabbit IgG Alexa Fluor 647, n.a., Invitrogen, A-31573, 1/2500
Fixable viability dye, n.a., eBioscience, 65-0866-18, 1/1000
Fixable viability dye, n.a., eBioscience, 65-0863-14, 1/1000

ANTIBODIES FOR CELL CULTURE
Antigen, Clone , Manufacturer, Catalog number
hCD3E, OKT3, Biolegend, 317326
mCD3E, 145-2C11, Biolegend, 100340
mCD28, 37.51, Biolegend, 102116
Armenian Hamster IgG Isotype Control, eBio299Arm, eBioscience, 16-4888-81
Goat anti-mouse IgG, Poly4053, Biolegend, 405301

ANTIBODIES FOR IMMUNOFLUORESCENCE/IMMUNOHISTOCHEMISTRY
Antigen, Clone , Manufacturer, Catalog, Dilution
TRAF3IP2 (ACT1), 9ACT12, Thermo Fisher Scientific, 14-4040-82, 1/200
GFP, D5.1, Cell Signaling, 2956S, 1/200

CD31, MEC 13.3, BD Biosciences, 550274, 1/50
NF-kB p65, D14E12, Cell Signaling, 8242S, 1/200
RELB, D7D7W, Cell Signaling, 10544S, 1/400
BCL-XL, 54H6, Cell Signaling, 2764S, 1/200
LCK, 73A5, Cell Signaling, 2787S, 1/200
CD3E, 17A2, Biolegend, 100223, 1/500
Donkey anti-rabbit IgG Alexa Fluor 647, n.a., Invitrogen, A-31573, 1/500
Goat anti-rat IgG Alexa Fluor 555, n.a., Invitrogen, A-21434, 1/500
Goat anti-rat IgG Alexa Fluor 647, n.a., Invitrogen, A-21247, 1/500
Donkey anti-rabbit IgG Rhodamine Red-X, n.a., Jackson ImmunoResearch, 711-295-152, 1/300

ANTIBODIES FOR WESTERN BLOT
Antigen, Clone , Manufacturer, Catalog, Dilution
TRAF3IP2 (ACT1), 9ACT12, Thermo Fisher Scientific, 14-4040-82, 1/1000 for WB and 10 µg/ml for co-IP
FYN, 15, Santa Cruz Biotechnologies, sc-434, 1/200
p65, D14E12, Cell Signaling, 8242S, 1/2000
Phospho-p65 (Ser536), 93H1, Cell Signaling, 3033S, 1/1000
p100/p52, polyclonal, Cell Signaling, 4882S, 1/1000
Phospho-p100 (Ser866/870), polyclonal, Cell Signaling, 4810S, 1/1000
ERK1/2, C-16, Santa Cruz Biotechnologies, sc-93, 1/1000
Phospho-pERK1/2 (Thr202/Tyr204), polyclonal, Cell Signaling, 9101S, 1/1000
JNK, polyclonal, Cell Signaling, 9252S, 1/1000
Phospho-JNK (Thr183/Tyr185), G9, Cell Signaling, 9255S, 1/1000
p38, A-12, Santa Cruz Biotechnologies, sc-7972, 1/1000
Phospho-p38 (Thr180/Tyr182), 3D7, Cell Signaling, 9215S, 1/1000
TRAF6, D21G3, Cell Signaling, 8028S, 1/1000 for WB and 1/50 for co-IP
K63-linked polyubiquitin, D7A11, Cell Signaling, 5621S, 1/1000
CARD11, 1D12, Cell Signaling, 4435S, 1/2000
LCK, 73A5, Cell Signaling, 2787S, 1/1000
Phospho-LCK (Tyr394), polyclonal, Sigma, SAB4300118, 1/1000
Na,K-ATPase, polyclonal, Cell Signaling, 3010, 1/1000
GFP, B-2, Santa Cruz Biotechnologies, sc-9996, 1/1000
Beta-actin, AC-15, Sigma, A1978, 1/5000
ECL Rat IgG HRP-linked whole antibody, polyclonal, Cytiva, NA935, 1/5000
ECL Rabbit IgG HRP-linked whole antibody, polyclonal, Cytiva, NA934, 1/5000
ECL Mouse IgG HRP-linked whole antibody, polyclonal, Cytiva, NA931, 1/5000

Validation

ANTIBODIES FOR FLOW CYTOMETRY
Antigen, Clone , Manufacturer, Catalog number, Dilution
CD3E, 17A2, Miltenyi Biotec, 130-118-849: QC tested (https://www.miltenyibiotec.com/BE-en/products/cd3e-antibody-anti-mouse-17a2.html#vioblue:30-ug-in-200-ul)
CD4, RM4-5, eBioscience, 46-0042-82: This RM4-5 antibody has been tested by flow cytometric analysis of mouse spleen cells (https://www.thermofisher.com/antibody/product/CD4-Antibody-clone-RM4-5-Monoclonal/46-0042-82)
CD4, RM4-5, eBioscience, 45-0042-82: This RM4-5 antibody has been tested by flow cytometric analysis of mouse thymocytes and splenocytes (https://www.thermofisher.com/antibody/product/CD4-Antibody-clone-RM4-5-Monoclonal/45-0042-82)
CD4, GK1.5, eBioscience, 25-0041-81: This GK1.5 antibody has been tested by flow cytometric analysis of mouse thymocytes and splenocytes (https://www.thermofisher.com/antibody/product/CD4-Antibody-clone-GK1-5-Monoclonal/25-0041-82)
CD4, GK1.5, eBioscience, 17-0041-82: The GK1.5 antibody has been tested by flow cytometric analysis of mouse thymocytes and splenocytes (https://www.thermofisher.com/antibody/product/CD4-Antibody-clone-GK1-5-Monoclonal/17-0041-82)
CD8, 53-6.7, eBioscience, 47-0081-82: This 53-6.7 antibody has been tested by flow cytometric analysis of mouse splenocytes (https://www.thermofisher.com/antibody/product/CD8a-Antibody-clone-53-6-7-Monoclonal/47-0081-82)
CD11b, M1/70, Biolegend, 101226: reactivity mouse, human (https://www.biolegend.com/en-us/products/apc-cyanine7-anti-mouse-human-cd11b-antibody-3930)
CD11b, M1/70, eBioscience, 69-0112-82: This M1/70 antibody has been tested by flow cytometric analysis of mouse bone marrow cells (https://www.thermofisher.com/antibody/product/CD11b-Antibody-clone-M1-70-Monoclonal/69-0112-82)
CD11b, M1/70, eBioscience, 12-0112-83: The M1/70 antibody has been tested by flow cytometric analysis of mouse splenocytes or bone marrow cells (https://www.thermofisher.com/antibody/product/CD11b-Antibody-clone-M1-70-Monoclonal/12-0112-82)
CD16/32, 93, eBioscience, 14-0161-86: The 93 antibody has been tested by flow cytometric analysis of mouse splenocytes (https://www.thermofisher.com/antibody/product/CD16-CD32-Antibody-clone-93-Monoclonal/14-0161-82)
CD19, eBio1D3, eBioscience, 25-0193-81: This eBio1D3 (1D3) antibody has been tested by flow cytometric analysis of mouse splenocytes (https://www.thermofisher.com/antibody/product/CD19-Antibody-clone-eBio1D3-1D3-Monoclonal/25-0193-82)
CD25, PC61.5, eBioscience, 17-0251-82: The PC61.5 antibody has been tested by flow cytometric analysis of mouse splenocytes (https://www.thermofisher.com/antibody/product/CD25-Antibody-clone-PC61-5-Monoclonal/17-0251-82)
CD44, IM7, BD Biosciences, 560570: Reactivity mouse (QC Testing) (https://www.bdbiosciences.com/us/applications/research/t-cell-immunology/t-follicular-helper-tfh-cells/surface-markers/mouse/percp-cy55-rat-anti-mouse-cd44-im7/p/560570)
CD69, H1.2F3, eBioscience, 25-0691-81: This H1.2F3 antibody has been tested by flow cytometric analysis of resting and activated mouse splenocytes (https://www.thermofisher.com/antibody/product/CD69-Antibody-clone-H1-2F3-Monoclonal/25-0691-82)
CD95, Jo2, BD Biosciences, 562633: Reactivity mouse (QC Testing) (https://www.bdbiosciences.com/us/applications/research/t-cell-immunology/regulatory-t-cells/surface-markers/mouse/bv421-hamster-anti-mouse-cd95-jo2/p/562633)
CD138, 281-2, Biolegend, 142503: reactivity mouse (https://www.biolegend.com/en-us/products/pe-anti-mouse-cd138-syndecan-1-

antibody-7519)
CD185/CXCR5, L138D7, Biolegend, 145506: Reactivity mouse (https://www.biolegend.com/en-us/products/apc-anti-mouse-cd185-cxcr5-antibody-8456)
CD278/ICOS, C398.4A, Biolegend, 313508: Reactivity Human, African Green, Baboon, Cynomolgus, Mouse, Rat, Rhesus, Swine (https://www.biolegend.com/de-at/products/pe-anti-human-mouse-rat-cd278-icos-antibody-2482)
CD278/ICOS, C398.4A, BD Biosciences, 565885: Reactivity Human (QC Testing) Mouse (Tested in Development) (https://www.bdbiosciences.com/eu/reagents/research/antibodies-buffers/immunology-reagents/anti-human-antibodies/cell-surface-antigens/buv395-armenian-hamster-anti-icos-cd278-c3984a/p/565885)
CD279/PD-1, 29F.1A12, Biolegend, 135218: Reactivity mouse (https://www.biolegend.com/en-us/products/brilliant-violet-421-anti-mouse-cd279-pd-1-antibody-7330)
CD279/PD-1, 29F.1A12, Biolegend, 135206: Reactivity mouse (https://www.biolegend.com/en-us/search-results/pe-anti-mouse-cd279-pd-1-antibody-6170)
B220, RA3-6B2, Biolegend, 103224: Reactivity mouse, human (https://www.biolegend.com/en-us/products/apc-cyanine7-anti-mouse-human-cd45r-b220-antibody-1938)
GL7, GL7, Biolegend, 144617: Reactivity mouse, human (https://www.biolegend.com/fr-fr/products/apc-anti-mouse-human-gl7-antigen-t-and-b-cell-activation-marker-antibody-17248)
Gr1, RB6-8C5, eBioscience, 12-5931-82: The RB6-8C5 antibody has been tested by flow cytometric analysis of mouse bone marrow cells and splenocytes (https://www.thermofisher.com/antibody/product/Ly-6G-Ly-6C-Antibody-clone-RB6-8C5-Monoclonal/12-5931-82)
Gr1, RB6-8C5, eBioscience, 17-5931-82: The RB6-8C5 antibody has been tested by flow cytometric analysis of mouse bone marrow cells and splenocytes
(https://www.thermofisher.com/antibody/product/Ly-6G-Ly-6C-Antibody-clone-RB6-8C5-Monoclonal/17-5931-82)
TCRbeta, H57-597, eBioscience, 12-5961-82: The H57-597 antibody has been tested by flow cytometric analysis of mouse thymocytes and splenocytes (https://www.thermofisher.com/antibody/product/TCR-beta-Antibody-clone-H57-597-Monoclonal/12-5961-82)
BCL6, K112-91, BD Biosciences, 563363: Reactivity Human (QC Testing) Mouse (Tested in Development) (https://www.bdbiosciences.com/eu/applications/research/t-cell-immunology/t-follicular-helper-tfh-cells/intracellular-markers/cell-signalling-and-transcription-factors/human/bv421-mouse-anti-bcl-6-k112-91/p/563363), PMID 29398449, positive and negative controls in figure 6b.
TdT, 19-3, eBioscience, 17-5846-82: This 19-3 antibody has been tested by intracellular staining using the Foxp3/Transcription Factor Staining Buffer Set (cat. 00-5521) followed by flow cytometric analysis of mouse thymocytes (https://www.thermofisher.com/antibody/product/TdT-Monoclonal-Antibody-19-3-APC-eBioscience/17-5846-82), positive and negative controls in figure 10d.
Phospho-ERK1/2, MILAN8R, eBioscience, 17-9109-42: species reactivity human, mouse (https://www.thermofisher.com/antibody/product/Phospho-ERK1-2-Thr202-Tyr204-Antibody-clone-MILAN8R-Monoclonal/17-9109-42)
Phospho-p65, 93H1, Cell Signaling, 3033S: species reactivity human, mouse, rat, hamster, monkey, pig (https://www.cellsignal.com/products/primary-antibodies/phospho-nf-kb-p65-ser536-93h1-rabbit-mab/3033)
IkBa, E130, Abcam, ab32518: reacts with mouse, rat, human (https://www.abcam.com/ikb-alpha-antibody-e130-ab32518.html)

The phospho-ZAP70 (n3kobu5), phospho-JNK (N9-66) and phospho-p38 (36/p38) antibodies have references on their use in murine cell lines on the manufacturer's websites, but the manufacturer only performed quality control testing in human cells. We validated these antibodies in murine T cells by incorporation of unstimulated and stimulated controls and by using FMO controls.

ANTIBODIES FOR CELL CULTURE
hCD3E, OKT3, Biolegend, 317326: Reactivity human (https://www.biolegend.com/en-us/products/purified-anti-human-cd3-antibody-3642?GroupID=GROUP28)
mCD3E, 145-2C11, Biolegend, 100340: Reactivity mouse (https://www.biolegend.com/en-us/products/ultra-leaf-purified-anti-mouse-cd3epsilon-antibody-7722)
mCD28, 37.51, Biolegend, 102116: Reactivity mouse (https://www.biolegend.com/en-us/products/ultra-leaf-purified-anti-mouse-cd28-antibody-7733)

ANTIBODIES FOR IMMUNOFLUORESCENCE/IMMUNOHISTOCHEMISTRY
TRAF3IP2 (ACT1), 9ACT12, Thermo Fisher Scientific, 14-4040-82: This 9ACT12 antibody has been tested by immunoblot of human peripheral blood cell lysates (https://www.thermofisher.com/antibody/product/ACT1-Antibody-clone-9ACT12-Monoclonal/14-4040-82)

GFP, D5.1, Cell Signaling, 2956S (https://www.cellsignal.com/products/primary-antibodies/gfp-d5-1-rabbit-mab/2956)
CD31, MEC 13.3, BD Biosciences, 550274: reactivity mouse (QC testing) (https://www.bdbiosciences.com/us/applications/research/stem-cell-research/cancer-research/mouse/purified-rat-anti-mouse-cd31-mec-133/p/550274)
NF-kB p65, D14E12, Cell Signaling, 8242S: species reactivity human, mouse, rat, hamster, monkey, dog (https://www.cellsignal.com/products/primary-antibodies/nf-kb-p65-d14e12-xp-rabbit-mab/8242)
RELB, D7D7W, Cell Signaling, 10544S: species reactivity human, mouse, rat (https://www.cellsignal.com/products/primary-antibodies/relb-d7d7w-rabbit-mab/10544)
BCL-XL, 54H6, Cell Signaling, 2764S: species reactivity human, mouse, rat, monkey (https://www.cellsignal.com/products/primary-antibodies/bcl-xl-54h6-rabbit-mab/2764)
LCK, 73A5, Cell Signaling, 2787S: species reactivity human (https://www.cellsignal.com/products/primary-antibodies/lck-73a5-rabbit-mab/2787), reactivity towards mouse (e.g. PMID 25609840)
CD3E, 17A2, Biolegend, 100223: species reactivity mouse

ANTIBODIES FOR WESTERN BLOT
Antigen, Clone , Manufacturer, Catalog, Dilution
TRAF3IP2 (ACT1), 9ACT12, Thermo Fisher Scientific, 14-4040-82, 1/1000 for WB and 10 µg/ml for co-IP
FYN, 15, Santa Cruz Biotechnologies, sc-434: Anti-Fyn Antibody (15) is recommended for detection of Fyn p59 of mouse, rat and human origin (https://www.scbt.com/p/fyn-antibody-15)

p65, D14E12, Cell Signaling, 8242S: species reactivity human, mouse, rat, hamster, monkey, dog (https://www.cellsignal.com/products/primary-antibodies/nf-kb-p65-d14e12-xp-rabbit-mab/8242)
Phospho-p65 (Ser536), 93H1, Cell Signaling, 3033S: species reactivity human, mouse, rat, hamster, monkey, pig (https://www.cellsignal.com/products/primary-antibodies/phospho-nf-kb-p65-ser536-93h1-rabbit-mab/3033)
p100/p52, polyclonal, Cell Signaling, 4882S: species reactivity human, mouse, rat, monkey (https://www.cellsignal.com/products/primary-antibodies/nf-kb2-p100-p52-antibody/4882)
Phospho-p100 (Ser866/870), polyclonal, Cell Signaling, 4810S: species reactivity human, mouse (https://www.cellsignal.com/products/primary-antibodies/phospho-nf-kb2-p100-ser866-870-antibody/4810)
ERK1/2, C-16, Santa Cruz Biotechnologies, sc-93: e.g. PMID 25981615, PMID 26567849
Phospho-pERK1/2 (Thr202/Tyr204), polyclonal, Cell Signaling, 9101S: species reactivity Human, Mouse, Rat, Hamster, Monkey, Mink, D. melanogaster, Zebrafish, Bovine, Pig, C. elegans (https://www.cellsignal.com/products/primary-antibodies/phospho-p44-42-mapk-erk1-2-thr202-tyr204-antibody/9101)
JNK, polyclonal, Cell Signaling, 9252S: species reactivity Human, Mouse, Rat, Hamster, Monkey, Zebrafish, Bovine, S. cerevisiae (https://www.cellsignal.com/products/primary-antibodies/sapk-jnk-antibody/9252)
Phospho-JNK (Thr183/Tyr185), G9, Cell Signaling, 9255S: species reactivity Human, Mouse, Rat, Hamster, S. cerevisiae (https://www.cellsignal.com/products/primary-antibodies/phospho-sapk-jnk-thr183-tyr185-g9-mouse-mab/9255)

p38, A-12, Santa Cruz Biotechnologies, sc-7972: is recommended for detection of p38 alpha MAPK14 and p38β of mouse, rat and human origin (https://www.scbt.com/p/p38alpha-beta-antibody-a-12)

Phospho-p38 (Thr180/Tyr182), 3D7, Cell Signaling, 9215S: species reactivity Human, Mouse, Rat, Monkey, D. melanogaster, Pig, S. cerevisiae (https://www.cellsignal.com/products/primary-antibodies/phospho-p38-mapk-thr180-tyr182-3d7-rabbit-mab/9215)

TRAF6, D21G3, Cell Signaling, 8028S: species reactivity Human, Monkey (https://www.cellsignal.com/products/primary-antibodies/traf6-d21g3-rabbit-mab/8028)

K63-linked polyubiquitin, D7A11, Cell Signaling, 5621S: all species (https://www.cellsignal.com/products/primary-antibodies/k63-linkage-specific-polyubiquitin-d7a11-rabbit-mab/5621); e.g. PMID 30770245, PMID 31519887
CARD11, 1D12, Cell Signaling, 4435S: species reactivity human, mouse (https://www.cellsignal.com/products/primary-antibodies/card11-1d12-rabbit-mab/4435)
LCK, 73A5, Cell Signaling, 2787S: species reactivity human (https://www.cellsignal.com/products/primary-antibodies/lck-73a5-rabbit-mab/2787), reactivity towards mouse (e.g. PMID 25609840)
Phospho-LCK (Tyr394), polyclonal, Sigma, SAB4300118: species reactivity rat, human, mouse (https://www.sigmaaldrich.com/catalog/product/sigma/sab4300118?lang=en&region=BE)
Na,K-ATPase, polyclonal, Cell Signaling, 3010: species reactivity human, mouse, rat, hamster, monkey, zebrafish (https://www.cellsignal.com/products/primary-antibodies/na-k-atpase-antibody/3010)
GFP, B-2, Santa Cruz Biotechnologies, sc-9996 (https://www.scbt.com/p/gfp-antibody-b-2)
Beta-actin, AC-15, Sigma, A1978, 1/5000: species reactivity human, mouse (https://www.sigmaaldrich.com/catalog/product/sigma/a1978)

Eukaryotic cell lines

Policy information about cell lines

Cell line source(s)	The Ba/F3 cell line was purchased from the DSMZ (ACC 300).<br>The Jurkat cell line was purchased from the DSMZ (ACC 282).<br>The 293T cell line was purchased from the DSMZ (ACC 635).
Authentication	Ba/F3: The identity of the cell line was confirmed by karyotyping.<br>Once per year we control the identity of Jurkat cells with STR profiling.<br>293T cells were not further authenticated.
Mycoplasma contamination	Cell lines were tested negative for mycoplasma on a regular basis (MycoAlert Mycoplasma Detection Kit, Westburg). Primary cells were cultured in Primocin (Invivogen, cat. code ant-pm-2), which contains 3 compounds with activity against mycoplasma species.
Commonly misidentified lines (See ICLAC register)	None

Animals and other organisms

Policy information about studies involving animals; ARRIVE guidelines recommended for reporting animal research

Laboratory animals	We used 8 to 10 week old C57BL/6J mice to harvest T cells for in vitro experiments. For bone marrow transplant experiments, we harvested lineage negative cells from 6 to 8 week old male C57BL/6J donor mice and we used 6 tot 8 week old female C57BL/6J mice as recipients. Mice were housed in individually ventilated cages with a temperature between 18 and 23°C and humidity between 40 and 60% No more than 5 mice were housed in a single cage. The room had a programmed 12 hour light-12 hour dark cycle.
Wild animals	The study did not involve wild animals.
Field-collected samples	The study did not involve field-collected samples.

Ethics oversight	Mouse experiments were approved and supervised by the KU Leuven ethical committee and conducted according to EU legislation (Directive 2010/63/EU).

Note that full information on the approval of the study protocol must also be provided in the manuscript.

Human research participants

Policy information about studies involving human research participants

Population characteristics	Age, gender, disease stage, IPI, prior therapy (boolean) are listed in supplementary table 1 for the discovery cohort. In addition, supplementary table 1 provides information on the clinical diagnostic immunophenotype of the lymphoma assessed by immunohistochemistry (CD3, CD4, CD8, GZMB, PFN, TIA1, CD30, ICOS, PD1, EBV) and mutational profile derived from RNA seq (TET2, IDH2, DNMT3A, RHOA). Supplementary table 2 specifies the PTCL subtype and immunophenotype assessed by immunohistochemistry (CD3, CD4, CD8, CD30, ICOS, PD1, CXCL13, Bcl6, CD10, PFN, TIA1, GZMB) for the validation cohort.
Recruitment	Patient samples for the discovery cohort were collected retrospectively from the tumor banks of the University Hospitals Leuven and the CHU Mont-Godinne and prospectively in the University Hospitals Leuven. For prospectively obtained samples, we obtained informed consent from all patients. Patient samples for the validation cohort were obtained from the T-cell lymphoma biobank (TENOMIC) of the Lymphoma Study Association (LYSA). Inclusion was based on the availability of sufficient material from a surgical biopsy. Therefore, patients with poor performance status or in critical condition that did not undergo a surgical biopsy, may have been missed due to the design of the study.
Ethics oversight	The study was approved by the Ethics Committee UZ/KU Leuven (S62100).

Note that full information on the approval of the study protocol must also be provided in the manuscript.

Flow Cytometry

Plots

Confirm that:

- [x] The axis labels state the marker and fluorochrome used (e.g. CD4-FITC).
- [x] The axis scales are clearly visible. Include numbers along axes only for bottom left plot of group (a 'group' is an analysis of identical markers).
- [x] All plots are contour plots with outliers or pseudocolor plots.
- [x] A numerical value for number of cells or percentage (with statistics) is provided.

Methodology

Sample preparation	Cultured cells were washed and resuspended in staining buffer (PBS with 2% FBS). Spleens and lymph nodes were smashed with the plunger of a 10 ml syringe on a 40 µm cell strainer. Pellets were incubated red blood cell lysis buffer (RCL, 150 mM NH_4Cl, 0.1 mM EDTA, 10 mM $KHCO_3$) and resuspended in staining buffer. Bones were flushed with medium, red blood cells lysed with RCL and pellets resuspended in staining buffer. Whole blood was lysed with RCL and pellets resuspended in staining buffer. Single cell suspensions were blocked with unconjugated CD16/32 antibody and stained with Fixable viability dye eFluor506 in staining buffer for 10 minutes protected from light at room temperature. Next cells were washed with stain buffer. For surface marker staining, cells were resuspended in staining buffer with the appropriate antibody dilutions for 30 minutes at 4°C protected from light.<br>For the detection of cytosolic proteins, cells were fixed with room temperature IC fixation buffer for 15 minutes and permeabilized with eBioscience Permeabilization buffer (both from ThermoFisher Scientific). For the detection of phosphorylated cytosolic proteins, cells were fixed with room temperature IC fixation buffer for 15 minutes and permeabilized with ice-cold methanol for 20 minutes. For the detection of phosphorylated transcription factors, cells were prepared with the Transcription Factor Buffer set (BD Biosciences) according to the manufacturer's instructions. For the detection of BCL6 and TdT, cells were processed with the FoxP3 transcription factor staining buffer set (ThermoFisher Scientific) according to the manufacturer's protocol. Cells were washed thoroughly and resuspended in permeabilization buffer or staining buffer (only methanol-permeabilized cells) with the appropriate antibody dilutions for 60 minutes or over night at 4°C protected from light. In case of unconjugated primary antibodies, cells were stained with secondary antibodies for 60 minutes at 4°C protected from light.
Instrument	Fortessa X-20 (Becton, Dickinson & Company Biosciences), FACSCanto II (Becton, Dickinson & Company Biosciences), FACSVerse (Becton, Dickinson & Company Biosciences), MACSQuant Vyb cytometer (Miltenyi), S3 cell sorter (Biorad), MA 900 cell sorter (Sony Biotechnology).
Software	Data was analyzed with FlowJo v10.6 (Becton, Dickinson & Company Biosciences).
Cell population abundance	Post-sort purity was verified with a MACSQuant Vyb cytometer (Miltenyi) for cultured cells. The purity of cells sorted prior to BCL6 staining was done with the Fortessa X-20 (Becton, Dickinson & Company Biosciences). Purity of the sorted populations was > 95%.

Gating strategy	All cells were gated on FSC-SSC profile to exclude debris. Next, we gated on single cells (FSC-W vs FSC-H). For all experiments - with the exception of the NF-kB GFP reporter cells and intracellular flow cytometry for phosphorylated proteins - cells were stained with a viability stain. We gated on viable, single cells for subsequent analyses.

☒ Tick this box to confirm that a figure exemplifying the gating strategy is provided in the Supplementary Information.
