# Supplementary material for: Fusion transcripts FYN-TRAF3IP2 and KHDRBS1-LCK hijack T cell receptor signaling in peripheral T-cell lymphoma, not otherwise specified
Source: Nat Commun. 2021 Jun 17;12:3705. doi: 10.1038/s41467-021-24037-4 (PMC8211700; doi:10.1038/s41467-021-24037-4)

Figure 2c

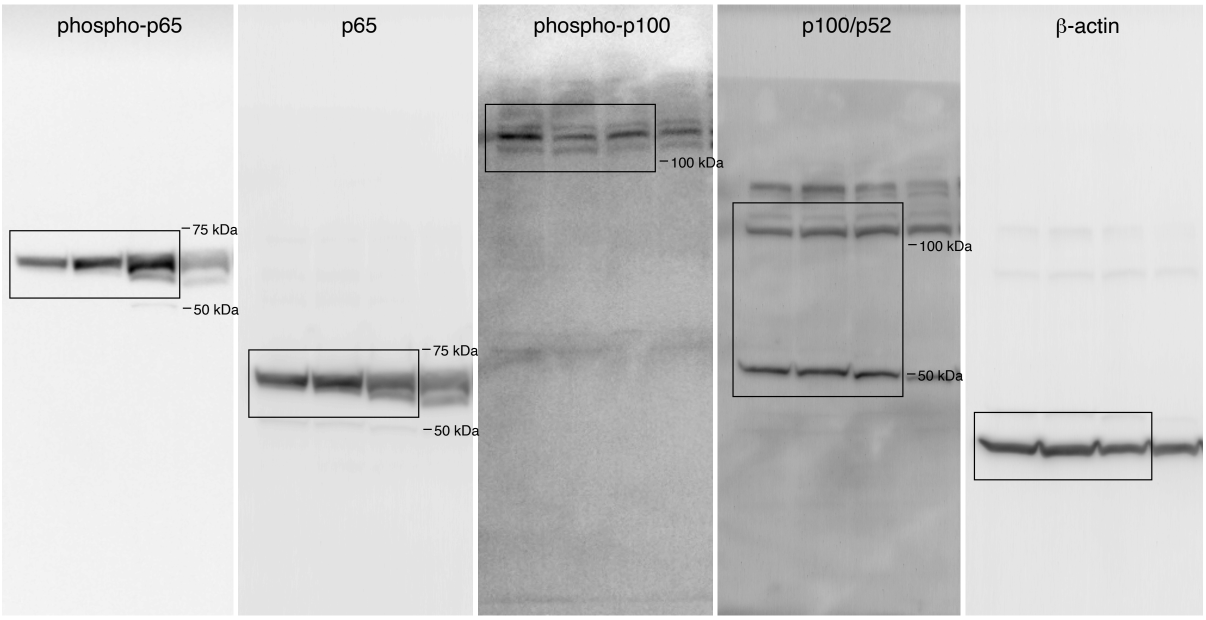

Figure 2e

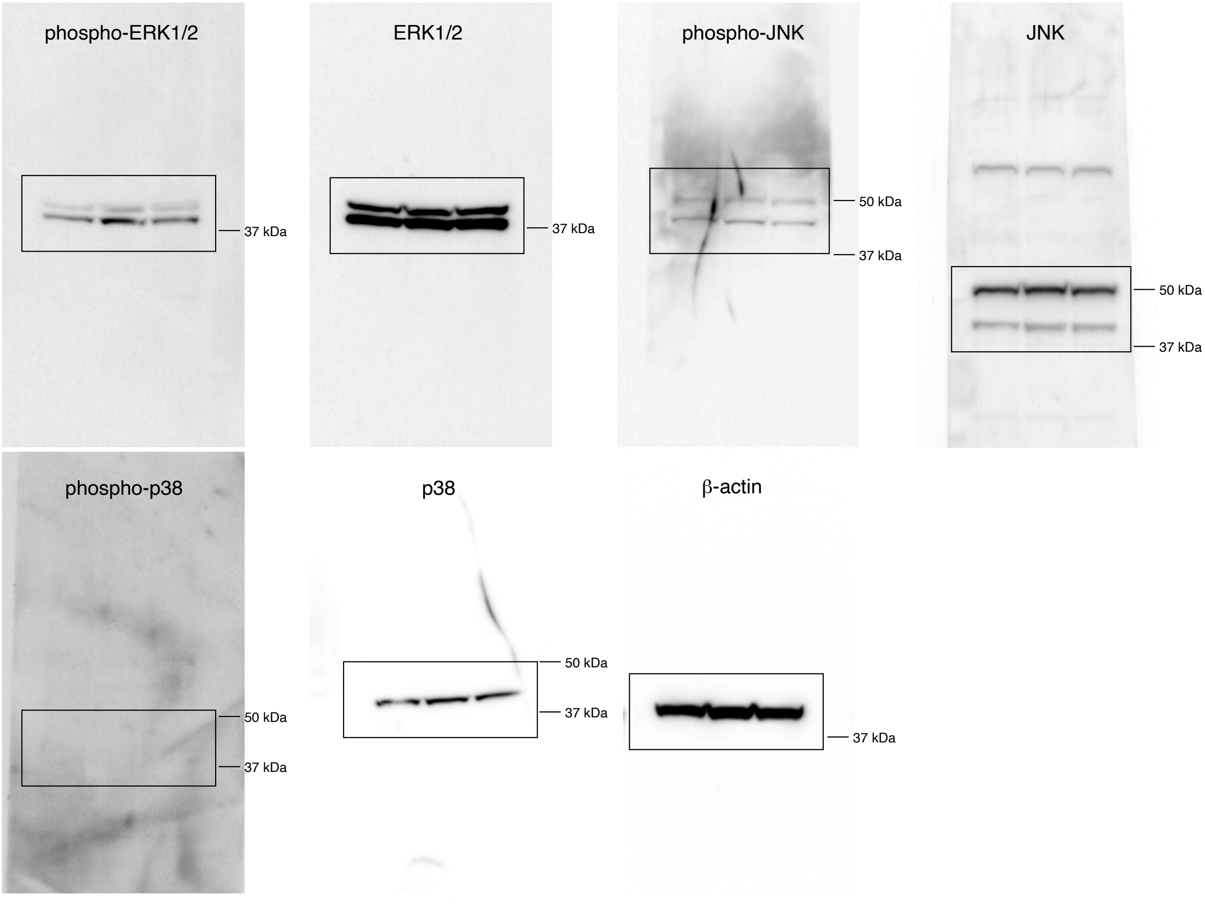

Figure 3c

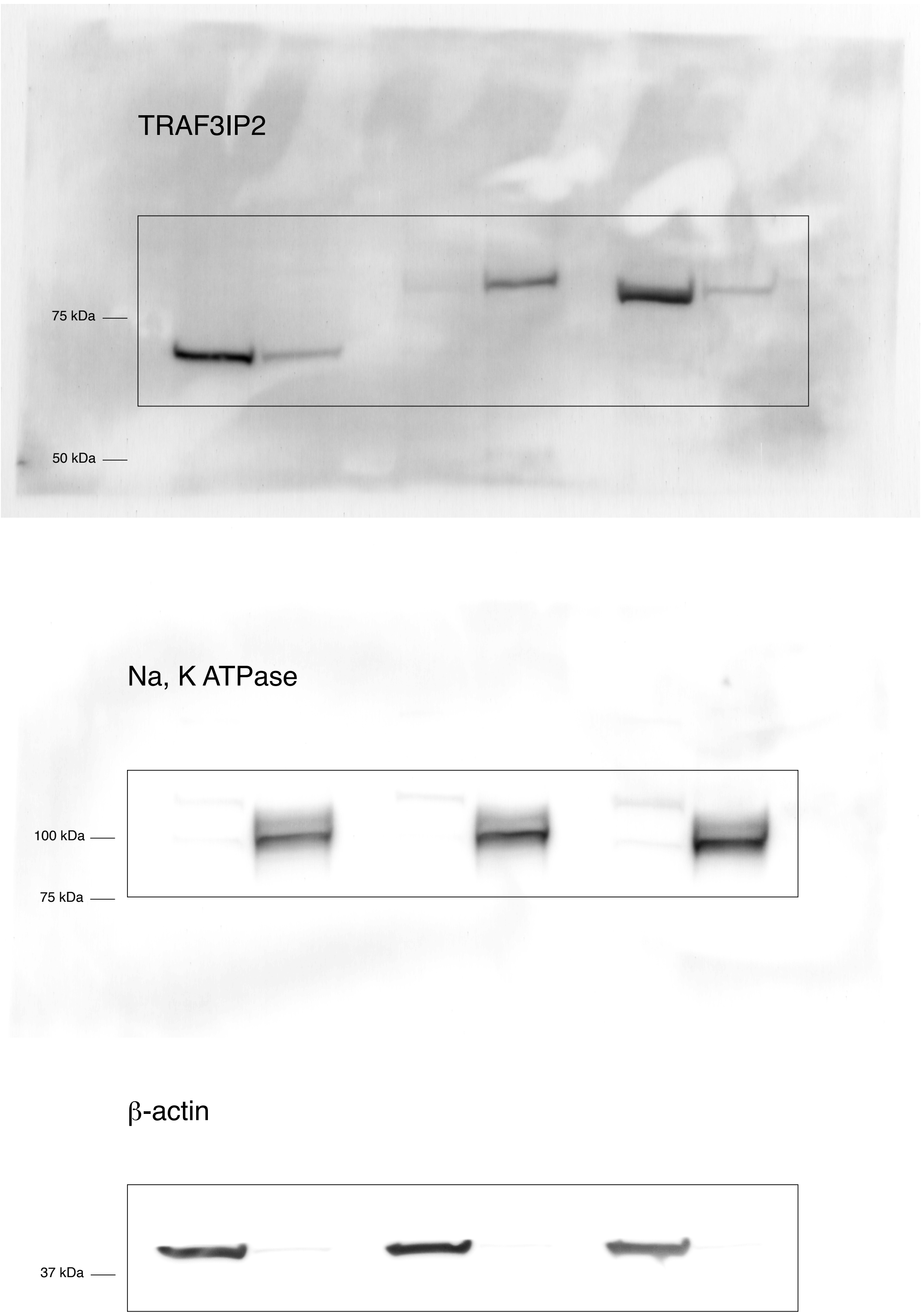

Figure 4a

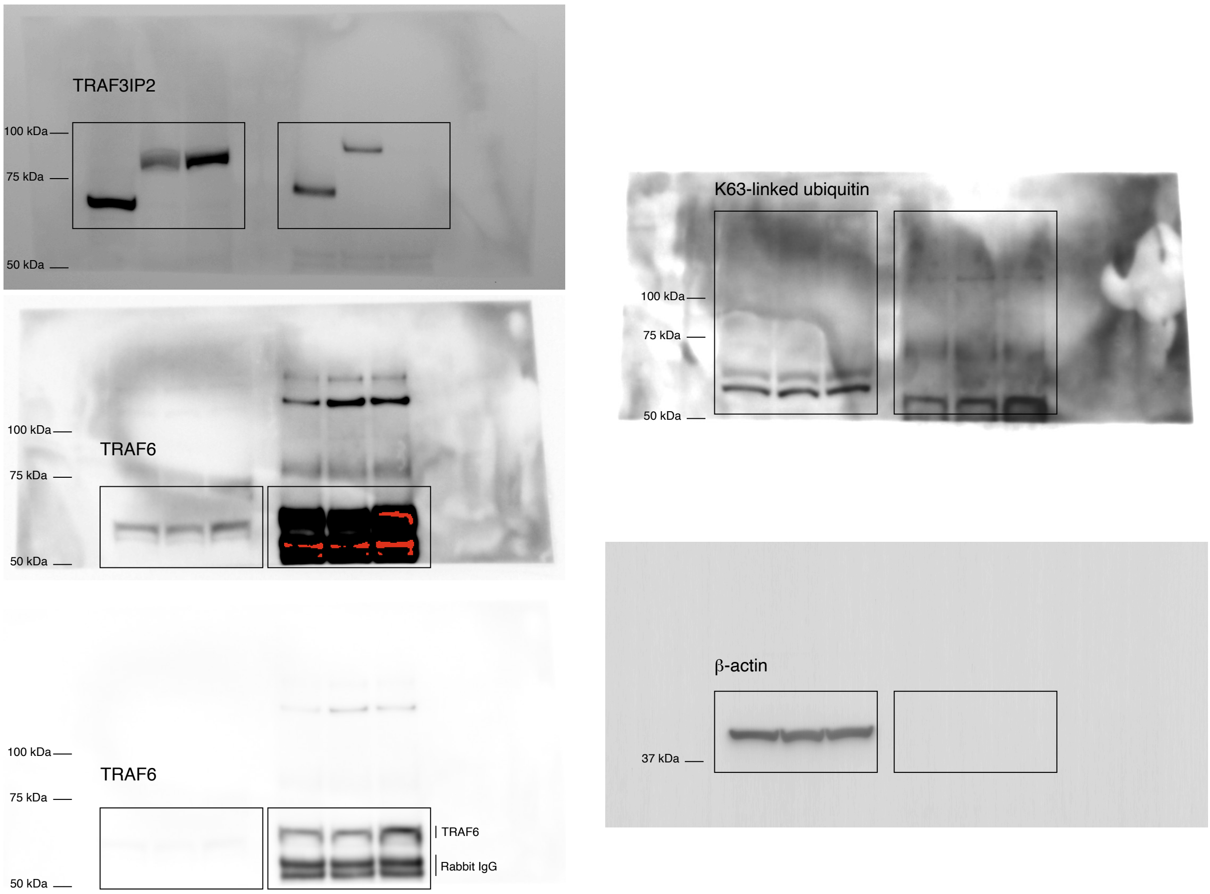
TRAF3IP2
100 kDa
75 kDa
50 kDa
TRAF6
100 kDa
75 kDa
50 kDa
TRAF6
100 kDa
75 kDa
50 kDa
TRAF6
Rabbit IgG
K63-linked ubiquitin
100 kDa
75 kDa
50 kDa
β-actin
37 kDa

Figure 4b

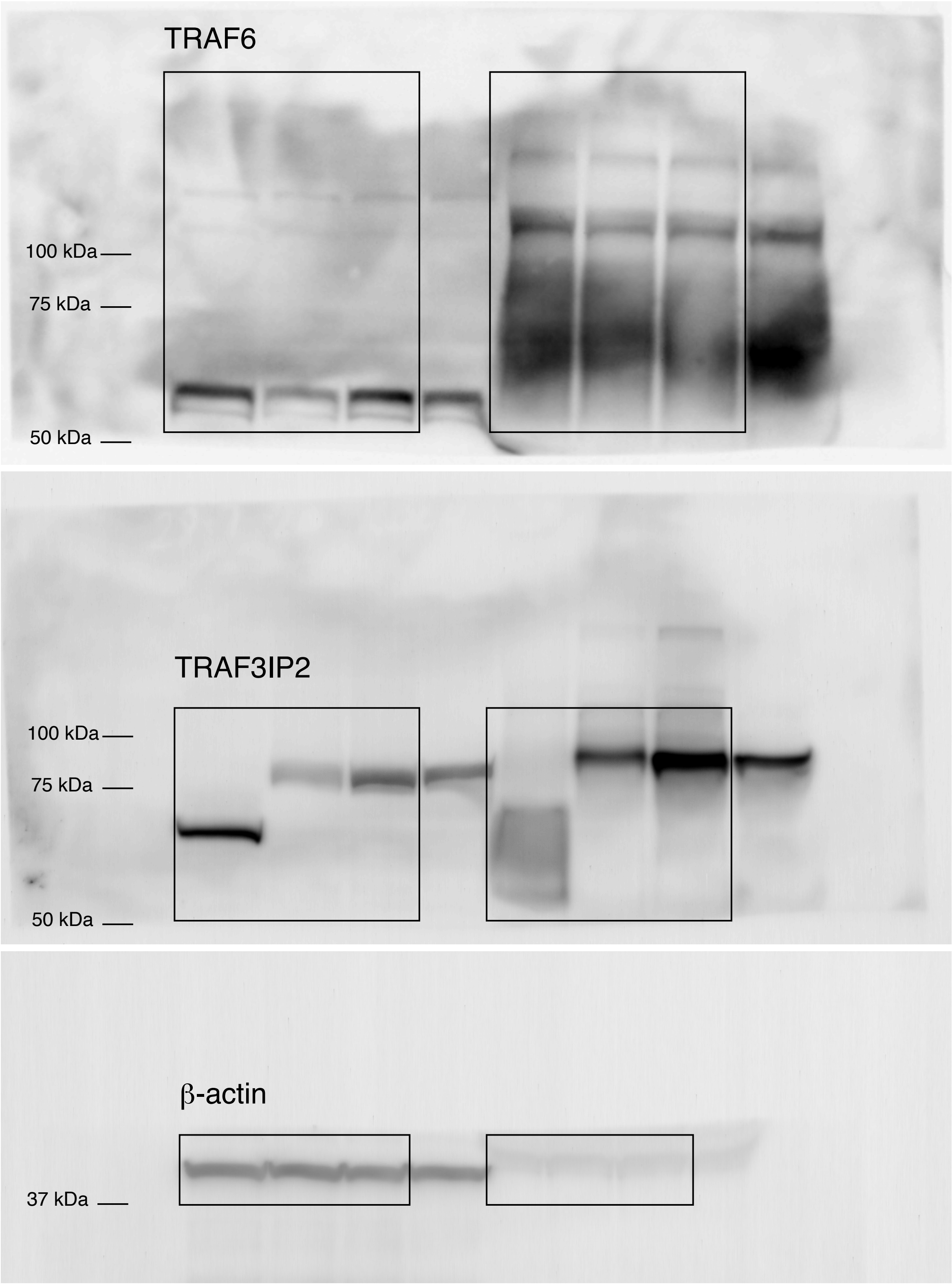
TRAF6
100 kDa
75 kDa
50 kDa
TRAF3IP2
100 kDa
75 kDa
50 kDa
β-actin
37 kDa

Figure 4d

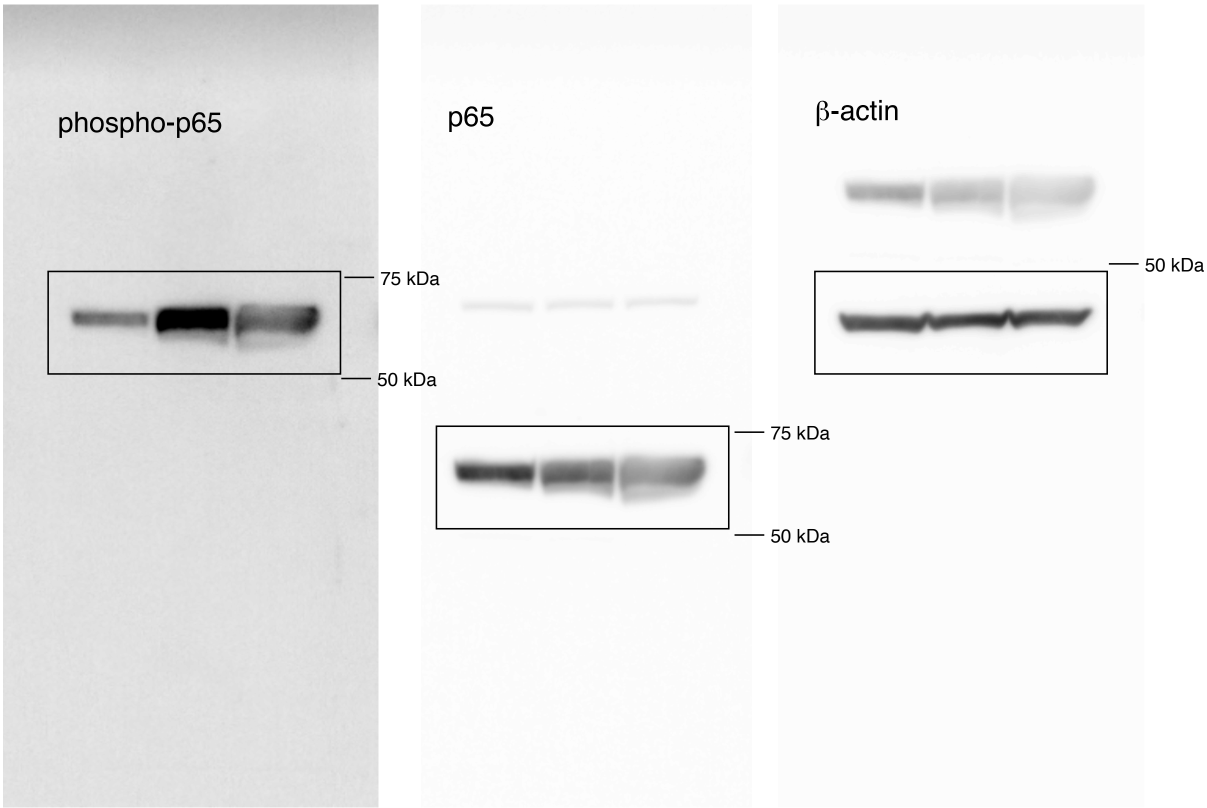

Figure 5g

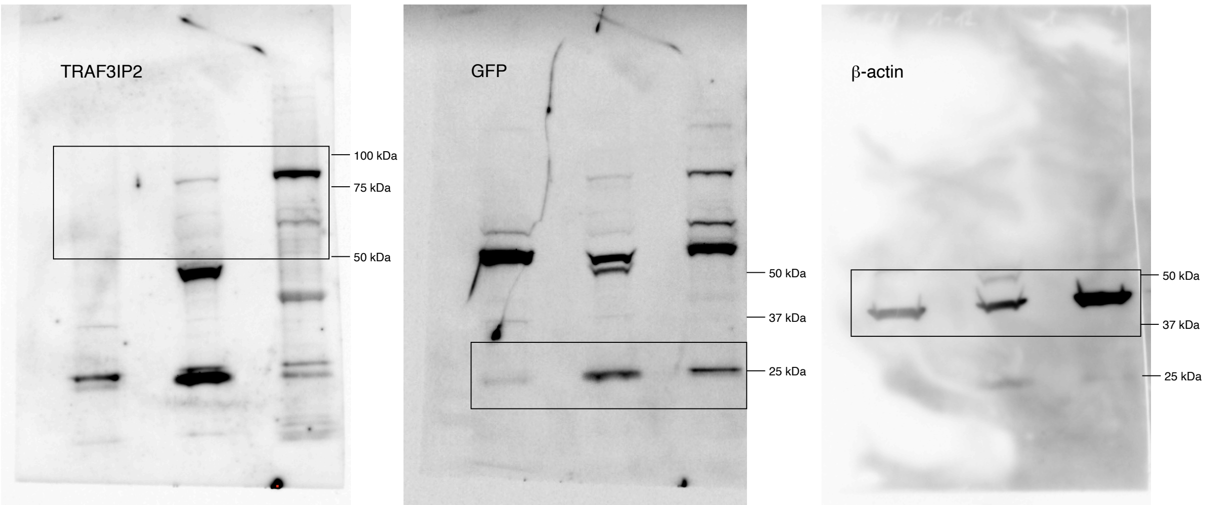

Figure 9d

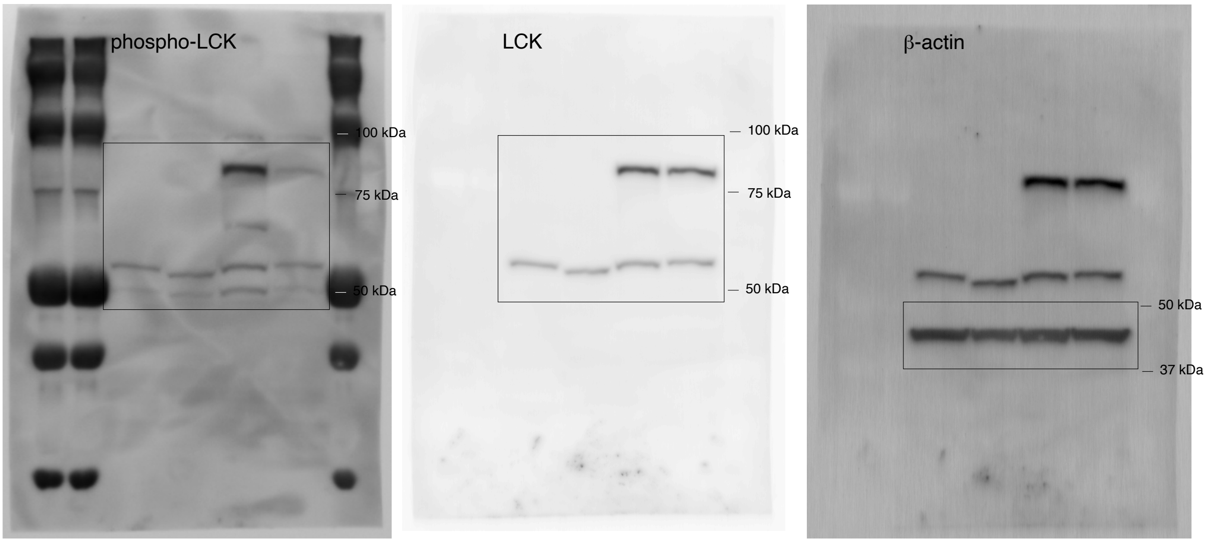

Figure 10e

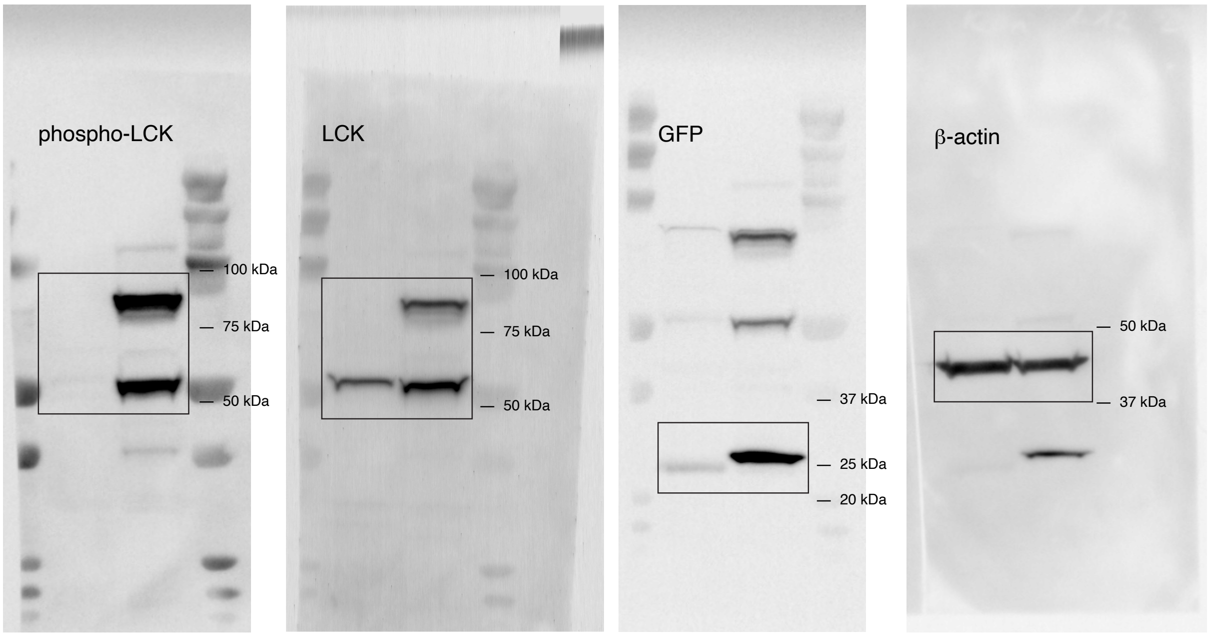

Supplementary figure 2a

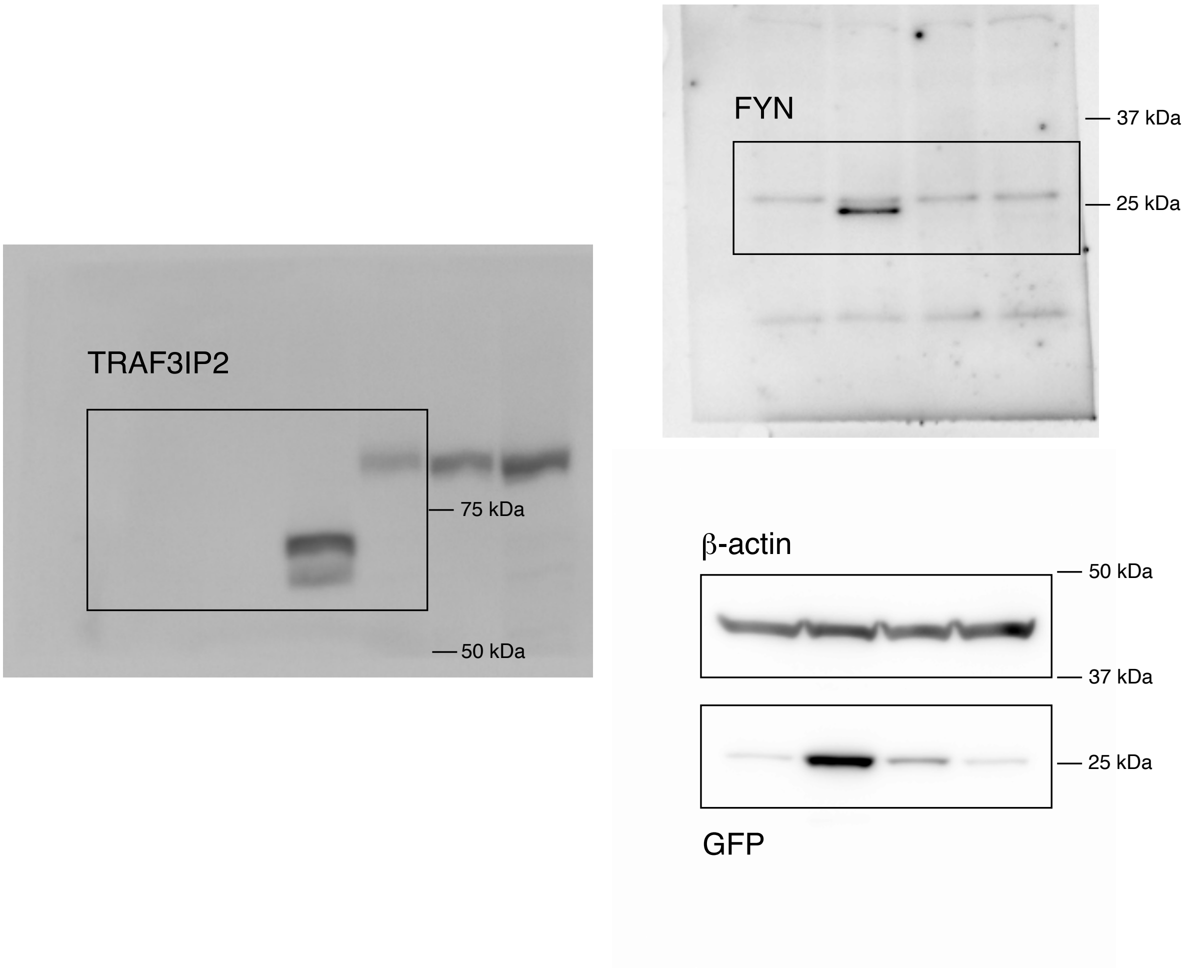

Supplementary figure 3a

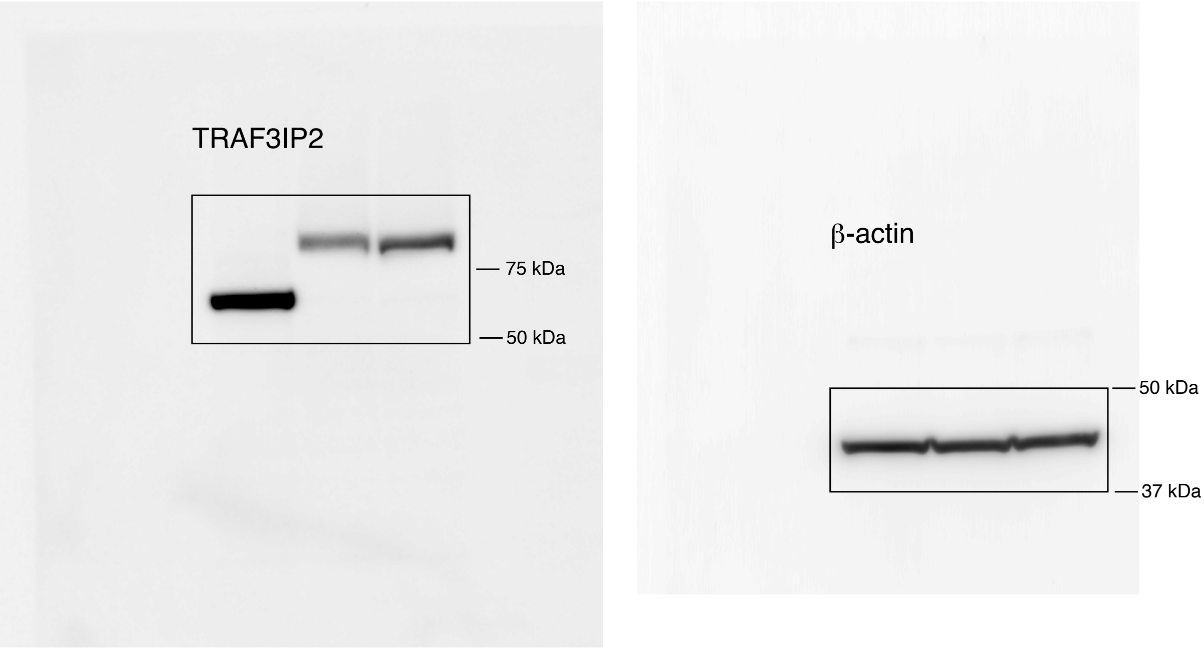

Supplementary figure 3b

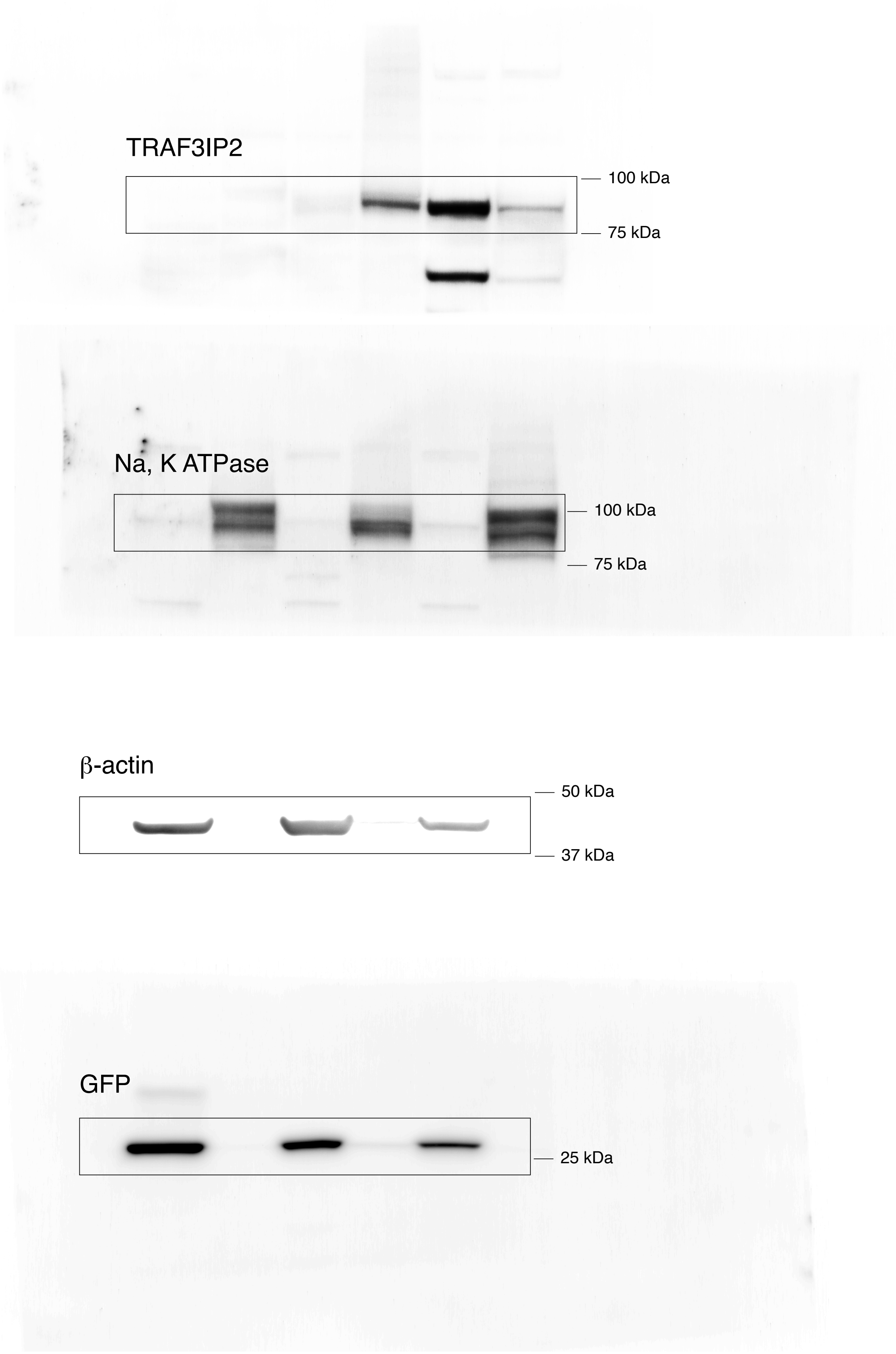

Supplementary figure 3c

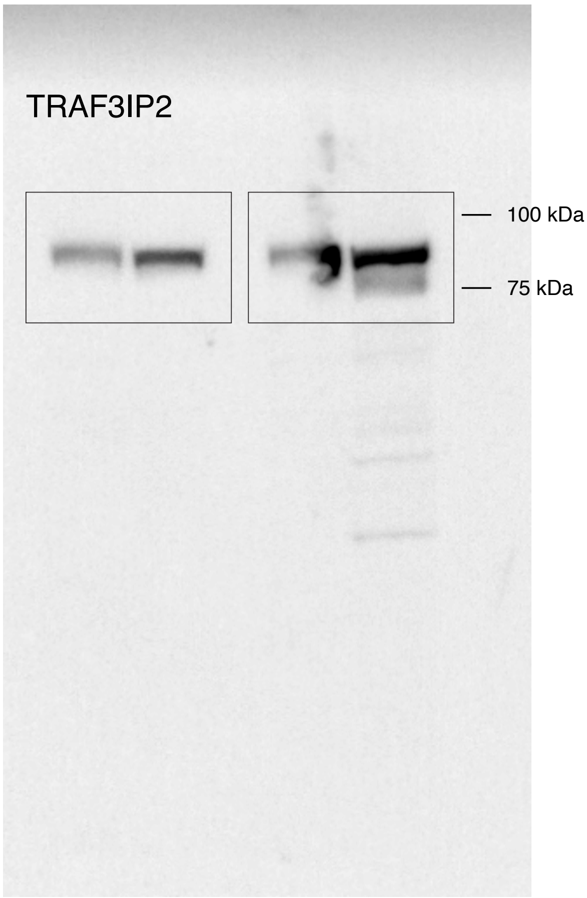

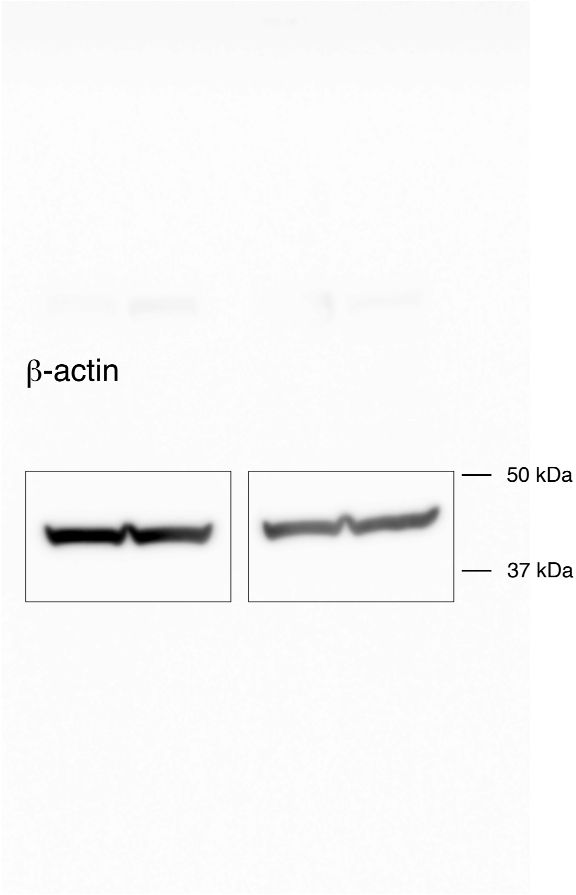

Supplementary figure 3d

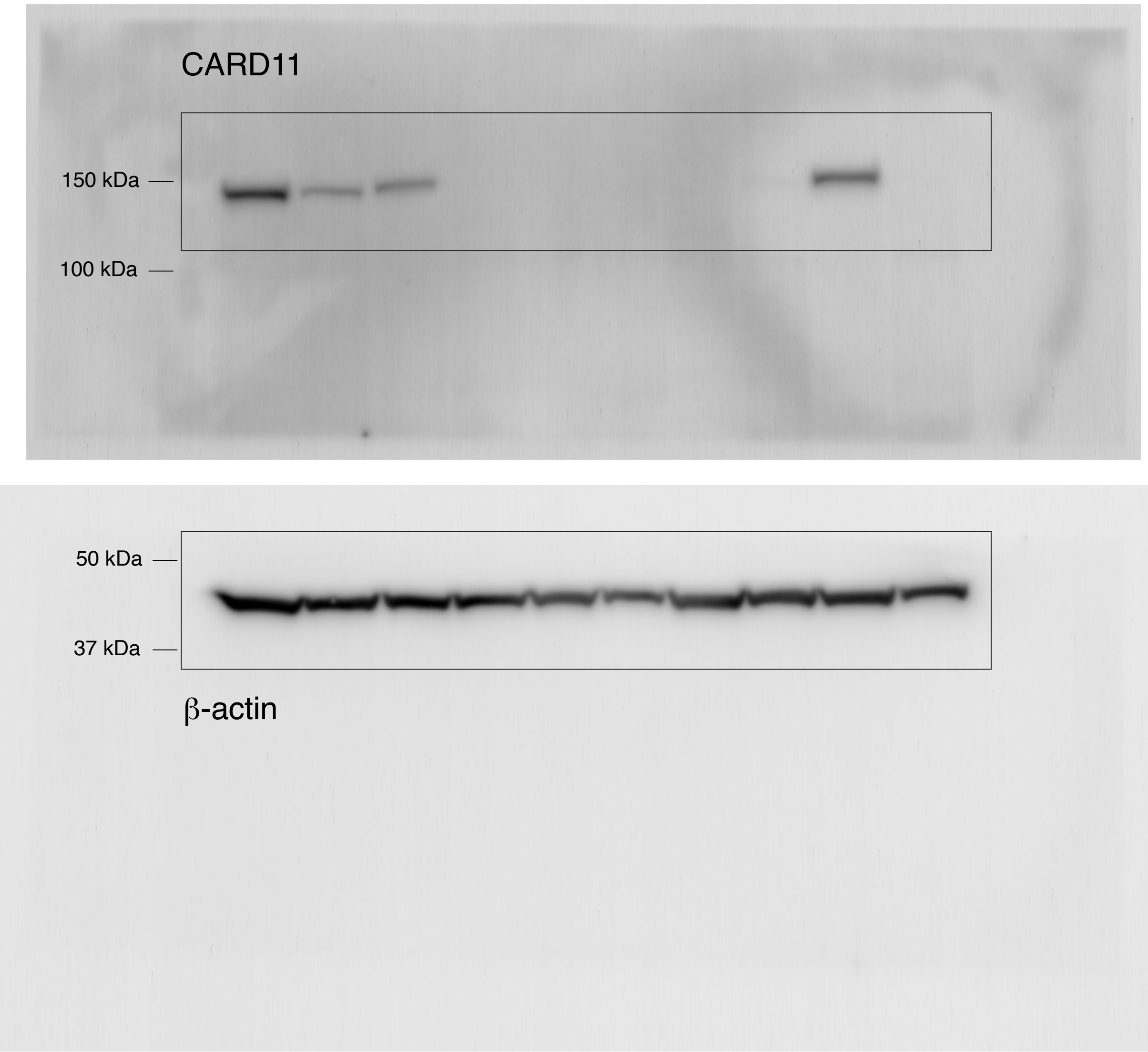

Supplementary figure 6a

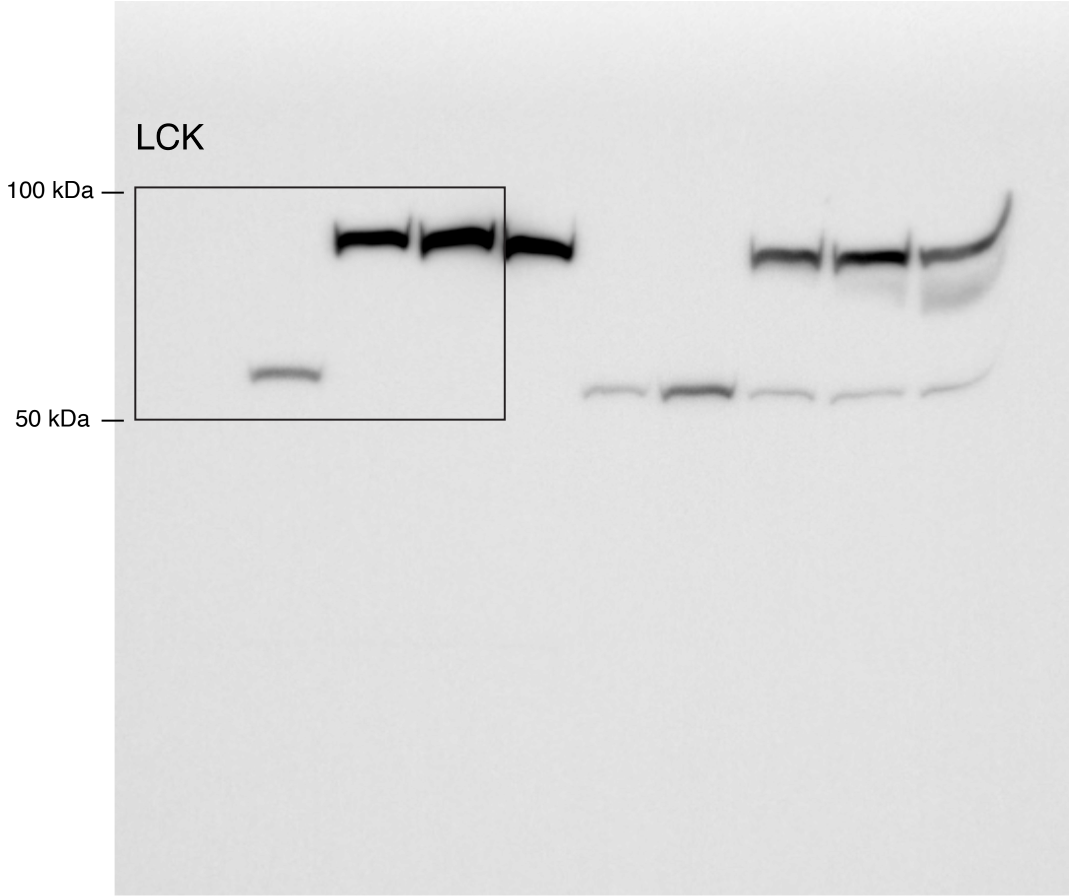

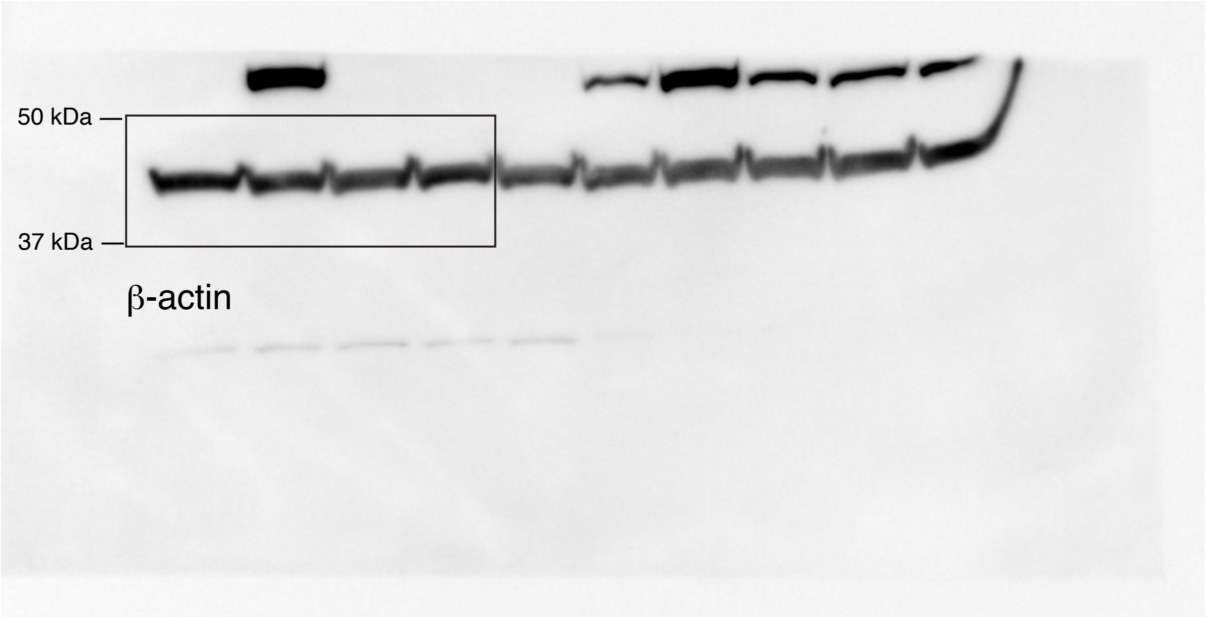

Supplement: Supplementary file 4 — Source Data [file 41467_2021_24037_MOESM4_ESM.zip › SourceData/SourceData_WB.pdf]
